# A Review of Dietary Intake of Acrylamide in Humans

**DOI:** 10.3390/toxics9070155

**Published:** 2021-06-30

**Authors:** Clara Amalie Gade Timmermann, Signe Sonne Mølck, Manik Kadawathagedara, Anne Ahrendt Bjerregaard, Margareta Törnqvist, Anne Lise Brantsæter, Marie Pedersen

**Affiliations:** 1 Department of Public Health, University of Copenhagen, 1356 Copenhagen, Denmark; signem89@hotmail.com; 2 Inserm, Institut de Recherche en Santé, Environnement et Travail, 35000 Rennes, France; manik.kadawathagedara@inserm.fr; 3 Center for Clinical Research and Prevention, Bispebjerg & Frederiksberg Hospital, 2000 Frederiksberg, Denmark; anne@ssi.dk; 4 Department of Epidemiology Research, Statens Serum Institute, 2300 Copenhagen, Denmark; 5 Department of Environmental Science, Stockholm University, 10691 Stockholm, Sweden; margareta.tornqvist@aces.su.se; 6 Department of Environmental Health, Norwegian Institute of Public Health, 0213 Oslo, Norway; AnneLise.Brantsaeter@fhi.no

**Keywords:** acrylamide, children, diet, epidemiological studies, humans, surveys and questionnaires

## Abstract

The dietary intake of acrylamide (AA) is a health concern, and food is being monitored worldwide, but the extent of AA exposure from the diet is uncertain. The aim of this review was to provide an overview of estimated dietary intake. We performed a PubMed search identifying studies that used dietary questionnaires and recalls to estimate total dietary AA intake. A total of 101 studies were included, corresponding to 68 original study populations from 26 countries. Questionnaires were used in 57 studies, dietary recalls were used in 33 studies, and 11 studies used both methods. The estimated median AA intake ranged from 0.02 to 1.53 μg/kg body weight/day between studies. Children were represented in 25 studies, and the body-weight-adjusted estimated AA intake was up to three times higher for children than adults. The majority of studies were from Europe (*n* = 65), Asia (*n* = 17), and the USA (*n* = 12). Studies from Asia generally estimated lower intakes than studies from Europe and the USA. Differences in methods undermine direct comparison across studies. The assessment of AA intake through dietary questionnaires and recalls has limitations. The integration of these methods with the analysis of validated biomarkers of exposure/internal dose would improve the accuracy of dietary AA intake exposure estimation. This overview shows that AA exposure is widespread and the large variation across and within populations shows a potential for reduced intake among those with the highest exposure.

## 1. Introduction

**Figure 1:**
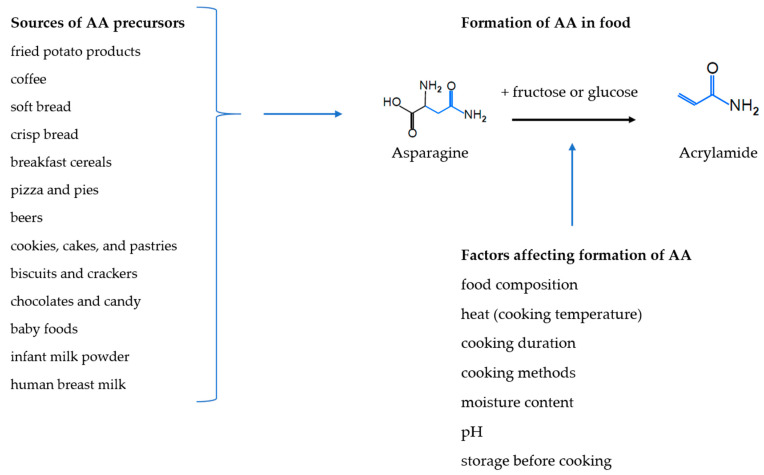
Sources, formation and factors affecting formation of acrylamide (AA).

In 2002 it was discovered that acrylamide (AA) forms in foods during heating, especially in foods rich in carbohydrates [1]. AA forms from sugars and amino acids mainly through the Maillard reaction that ‘browns’ and affects the taste of food during high-temperature processing [2,3] (Figure 1). AA is classified as a ‘*probable human carcinogen*’, carcinogenicity Group 2A by the International Agency of Research on Cancer (IARC) on the basis of its genotoxicity and carcinogenicity in rodents [4].

Food agencies in the European Union member states [5], Canada [6], the U.S. Food and Drug Agency (FDA) [7], Australia [8], New Zealand [9], and many other countries monitor a wide range of foods and drinks for AA, and the highest AA concentrations have been found in fried potato products and coffee [5,7], but other foods with lower concentrations that are consumed in higher amounts are also important sources of AA, such as soft bread, breakfast cereals, sweet bakery products, biscuits, crackers, and crisp bread [5,7,10]. AA has also been detected in homemade food, as well as in human breast milk [11], infant milk powder and a range of baby foods [5,10] (Figure 1). The dietary sources of AA vary between adults, children and infants [5].

The formation of AA depends mostly on the sugars, asparagine and other amino acids contents of the foods and the cooking temperature and duration, but it is also affected by storage methods, pH, and moisture content, as well as cooking methods [12,13,14] (Figure 1). The concentration of AA can, therefore, vary even within the same kinds of food items for instance in potato crisps where concentrations range from 0 to almost 2000 μg/kg [5]. European AA monitoring data show a decrease in AA concentrations in potato crisps from 2002 to 2011 [5], but changes in potato crisps AA concentrations were limited between 2007 and 2010, and concentrations in processed cereal-based infants foods have decreased between 2007 and 2010 [15]. AA concentration in coffee and coffee substitutes have been found to decrease between 2002 and 2015 [16] but to increase between 2007 and 2010 [15] and between 2016 and 2018 [16].

The individual dietary intake of AA also depends on dietary habits in terms of recipes, portion size and frequency of consumption. The overall dietary intake of AA and related heat-generated exposure, therefore, varies markedly between and within populations and across time. In 2011, the Joint Expert Committee on Food Additives (JECFA) by the World Health Organization (WHO) and the Food and Agriculture Organization (FAO) of the United Nations evaluated the dietary AA intake of children and adults from eight countries (i.e., Brazil, China, France, Ireland, New Zealand, Norway, Spain, and United Kingdom) and reported average national intakes of between 0.2 (Spain) to 1.0 (New Zealand) μg/kg body weight (bw)/day [17], though regional data on adolescents in Brazil estimated an average AA intake of only 0.1 μg/kg bw/day [17]. In a previous JECFA report from 2006, covering 17 countries (i.e., Australia, Belgium, Canada, China, Hong Kong, Czech Republic, Denmark, France, Germany, the Netherlands, New Zealand, Norway, Sweden, Switzerland, the United Arab Emirates, the United Kingdom, and the USA), the averages of AA intake were estimated to range between 0.3 (China, Hong Kong) and 2.0 (Children < 12 years in the United Arab Emirates) μg/kg bw/day [10]. Relative to their body mass, children consume a large amount of starchy foods, such as cereal and baked products and it has been estimated that the dietary AA intake of children is 2-3 times higher compared to adults [10,17]. In a 2015 report, based on European data, the European Food Safety Authority (EFSA) estimated average AA intakes as ranging between 0.4 and 1.9 μg/kg bw/day across all age groups and, in children, the average intake ranged between 0.5 and 1.9 μg/kg bw/day, whereas the average intake among adolescents, adults, the elderly, and very elderly were between 0.4 and 0.9 μg/kg bw/day [5]. Based on evidence from animal studies, the EFSA Panel on Contaminants in the Food Chain (CONTAM) agreed with previous evaluations that AA in food potentially increases the risk of developing cancer for humans in all age groups, and they noted that the high intake in children is of particular concern due to their expected longer lifespan ahead and the increased vulnerability during early life development and growth [5].

To date, the epidemiological evidence on the relationship between dietary intake of AA and cancer in humans is lacking from most cancer sites, but evidence of association with kidney cancer is accumulating [18] and increased risk of endometrial and ovarian cancers has also been suggested in non-smoking women when comparing high versus low dietary AA intake as defined in the original studies [18]. Studies of the risk of cancer and other adverse health effects associated with AA intake are challenged by the fact that the estimation of dietary intake of AA is uncertain [5,19]. Previous reviews on human dietary AA intake have focused on associations with cancer [19,20] while summarizing methods and data from 41 epidemiological studies [19]. To the best of our knowledge, the estimated dietary AA intake levels from total diet across different study populations have not previously been compiled in a peer reviewed publication. The aim of our review is thus to summarize the estimated total dietary intake of AA, as assessed through dietary questionnaire (DQ) and dietary recall/record (DR), and to compare the estimated intake across age groups and countries, where possible. We, thereby, aim to provide an overview of total dietary intake of AA across different populations. In addition, we will discuss existing data gaps, and give future study directions.

## 2. Methods

Original research publications in which AA intake from total diet in humans was estimated using DQ, including food frequency questionnaires and/or DR combined with AA levels from food monitoring, were eligible for our review. Furthermore, we restricted our review to studies published in peer-reviewed scientific journals. We excluded reviews, comments, and abstracts, as well as studies published in languages other than English. Since we were interested in total dietary intake, we also excluded studies evaluating AA intake following diet interventions and studies on selected meals or food items (e.g., only bread). Finally, we excluded studies estimating the internal dose of AA through analyses of biomarkers without reporting the estimated dietary intake of AA. Likewise, studies were excluded if dietary AA intake was estimated using only duplicate diets, in which AA was measured in a copy of the food and drinks consumed by the participants, with no data from DQs or DRs.

### 2.1. Search Strategy

We performed a systematic literature search on PubMed to identify studies that estimated the dietary intake of AA in humans from DQs or DRs. The final search was performed on 22 February 2021 and consisted of three facets (Table 1). The search terms within each facet were separated by an ‘OR’ operator, and the three facets were combined using ‘AND’ operators. We found additional studies through a chain search using references from the first identified studies and from the most recent report on AA from EFSA [5].

### 2.2. Selection and Data Collection Process

All abstracts were reviewed by at least one author (C.A.G.T., S.S.M. or M.P.) and those that were not excluded during the abstract screening were read in full (C.A.G.T., S.S.M. or M.P.). Studies excluded during the abstract screening or full text screening were reviewed by at least one additional author (C.A.G.T. or M.P.) to avoid the exclusion of potentially relevant studies.

For each of the included individual studies, at least one author abstracted information about the study population, size, geographic location, dietary method, years of dietary assessment, source or method of obtaining AA levels in foods, as well as the median and minimum–maximum values of the estimated dietary AA intake, if available. When medians were not given, we reported mean and standard deviation, geometric mean, medians or means of quartiles, or means of quintiles (in prioritized order). When minimum–maximum values were not given, we obtained information about the most extreme percentiles given. We obtained information about both bw-adjusted and non-bw-adjusted estimates, if given.

### 2.3. Synthesis Methods

When possible, we presented estimates for dietary AA intake separately for children and adults, and separately for different countries. However, other subgroups, including males and females, cases and controls, smokers and non-smokers, were only presented separately if no joint estimate was given. Medians of the estimated total dietary AA intake was summarized in bar graphs by countries and in studies also including children by age groups. When medians were not given, we used means. Separate graphs were created for bw-adjusted and non-bw-adjusted estimates. However, for studies on children, we did not present non-bw-adjusted estimates in graphs, as only three studies reported such estimates. Furthermore, separate graphs were created for studies using (1) DQs and DQs in combination with DRs; (2) studies using only DRs. When estimates from the same population were presented in more than one study, we used estimates from only one of the studies. We prioritized the study that was published first unless a later study covered more individuals or provided data that were more useful for our synthesis (e.g., joint estimates instead of gender stratified estimates).

## 3. Results

**Figure 2:**
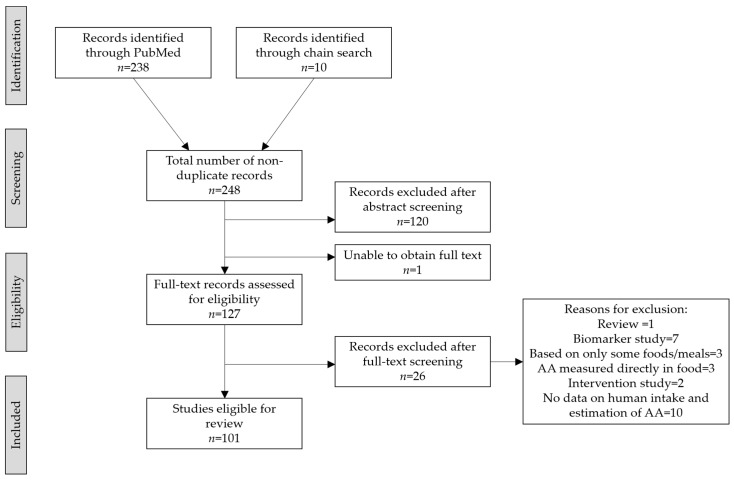
Prisma flowchart.

Our PubMed search resulted in 238 publications, and additionally 10 publications were identified through chain search (Figure 2). We excluded 120 studies (i.e., original publications) in the abstract screening and additionally 26 studies in the full text screening. We were unable to obtain access to the full text of one study as it was not available online, and libraries with hard copies were closed due to the COVID-19 lockdown. Thus, a total of 101 studies covering 26 countries were included (Supplementary Table S1).

### 3.1. Demography of Study Populations

The 101 studies included in this review were published between 2003 and 2020. One study included populations in both the USA, Australia, and Italy, while nine studies covered populations across European countries. Additionally, 56 studies were from a European country, 17 were from Asia, 12 were from the USA, and the remaining 6 were from Australia (*n* = 1), Iran (*n* = 1), Turkey (*n* = 1), Canada (*n* = 2), and Brazil (*n* = 1). Among the European studies, 17 were from the Netherlands, and 14 were from Sweden. However, many of the studies covered the same populations and, thus, we found data from three unique populations in the Netherlands and nine in Sweden. In total, the 101 studies covered 68 unique populations. When a study included populations from several countries, we counted each country as a population.

Information on intake of food and drinks were collected between years 1976 and 2018 and covered periods up to 5 years, as well as dietary intake up to 20 years back in time. However, the majority of DQs covered the preceding 1 year, while the majority of DRs covered the preceding 24–48 h (Supplementary Table S1). The majority of studies (92/101 corresponding to 61/68 unique populations) were based on data from adults aged 18 years or older, while children (<18 years) were represented in 25 studies (corresponding to 21 unique populations). Dietary AA intakes for pregnant women were estimated in six studies (corresponding to three unique populations) from Norway, France and Japan [21,22,23,24,25,26]. Not all studies reported the number of individuals included but, based on the reported data, 1,238,703 individuals were included across 63 study populations, and among these, 27,598 were children from across 18 study populations.

### 3.2. Estimated AA Intakes across Countries and Age-Groups

**Figure 3:**
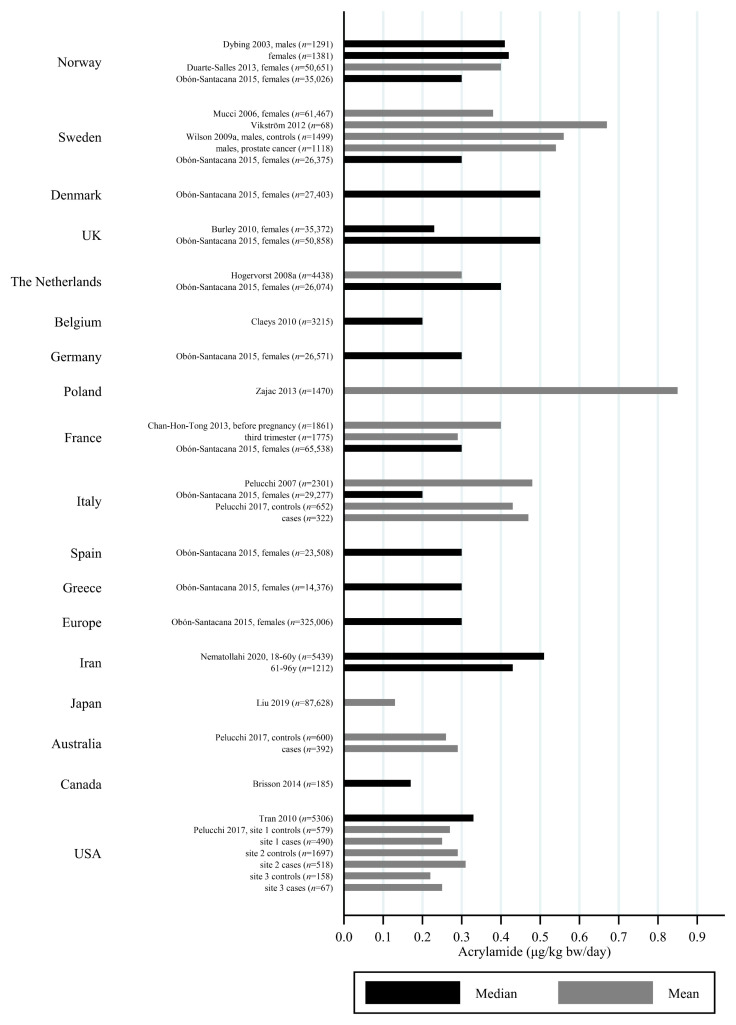
Median/mean estimated daily acrylamide intake per kg body weight (μg/kg bw/day), based on dietary questionnaire or both dietary questionnaire and dietary recall, by country.

**Figure 4:**
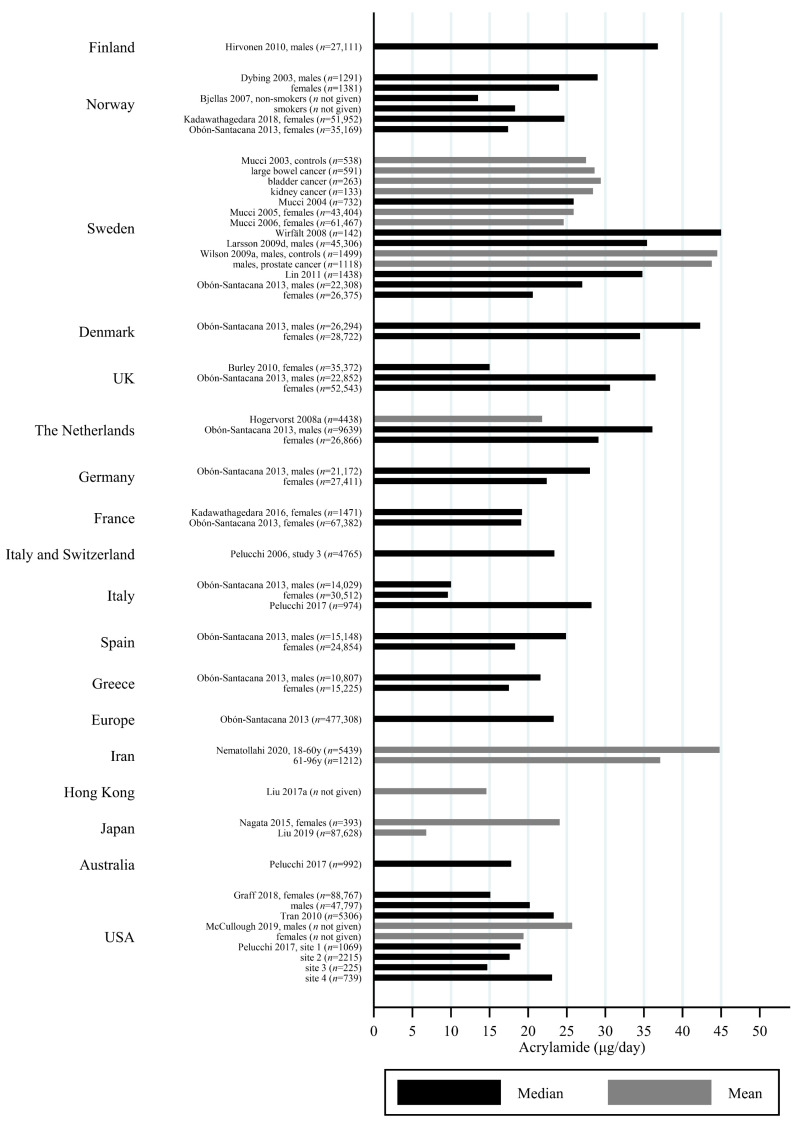
Median/mean estimated daily acrylamide intake (μg/day), based on dietary questionnaire or both dietary questionnaire and dietary recall, by country.

**Figure 5:**
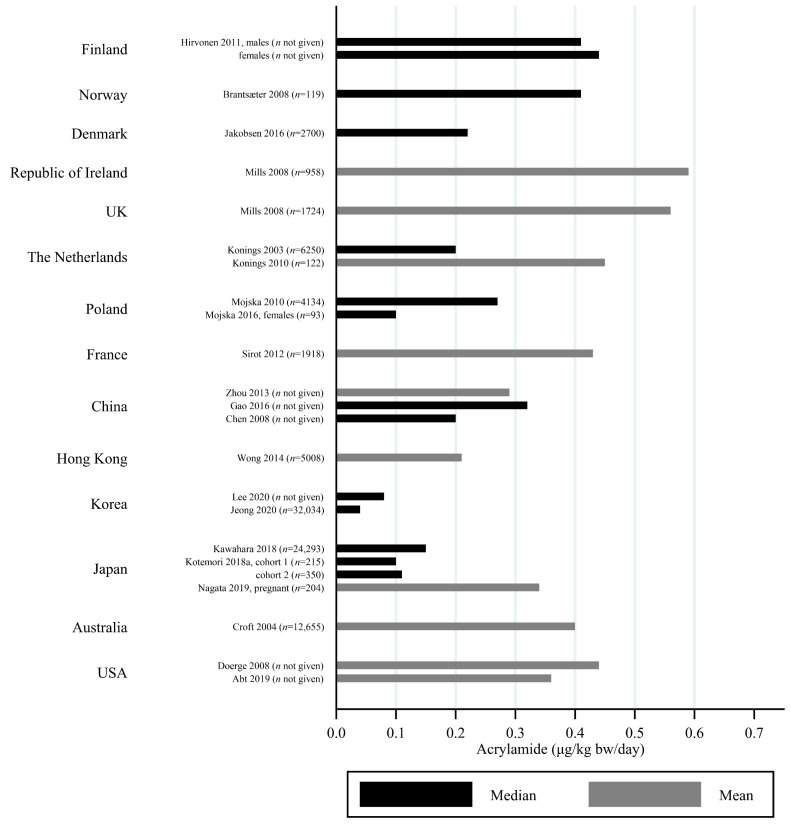
Median/mean estimated daily acrylamide intake per kg body weight (μg/kg bw/day), based on dietary recall, by country.

**Figure 6:**
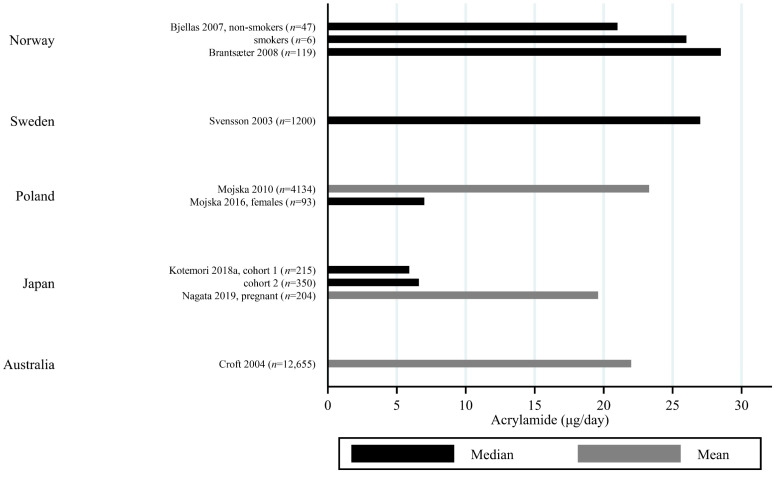
Median/mean estimated daily acrylamide intake (μg/day), based on dietary recall by country.

The estimated median dietary AA intake across all studies summarized in Supplementary Table S1 ranged from 5.9 μg/day among 45–74 year olds in Japan [27] to 45 μg/day in a Swedish study where the participants aged 45–73 years were specifically selected to obtain a wide variation in estimated AA intake [28]. The mean ranged from 6.8 μg/day among 40–69 year olds in Japan [29] to 55.1 μg/day among Iranian 11–17 year olds [30]. When adjusting for bw, the median ranged from 0.02 μg/kg bw/day among Korean 65–80 year olds [31] to 1.53 μg/kg bw/day among Iranian 3–10 year olds [30]. The mean ranged from 0.12 μg/kg bw/day among Japanese 60+-year-olds [32] to 1.68 μg/kg bw/day among Turkish 1 year olds [33]. Several studies estimated the minimum dietary intake of AA to be zero [34,35,36,37,38,39], while the same studies found maximum estimated dietary intake up to 261 μg/day [36] and 5.78 μg/kg bw/day [39]. The highest bw-adjusted estimated dietary intake (6.41 μg/kg bw/day) was found among Turkish toddlers [33]. Thus, large variation in estimated dietary AA intake was seen within studies. In comparison to the variation within each study a smaller variation was seen between studies within the same country, e.g., Sweden, Norway, the USA and Japan (Figure 3, Figure 4 and Figure 5). The studies that deviated the most from the country average/median, were based on selected groups, e.g., pregnant Japanese women [25] had higher estimated AA intake (mean 19.6 μg/day and 0.34 μg/kg bw/day) than the estimated intake of AA of the general Japanese population (Figure 5 and Figure 6), and Swedish hospital patients (mean AA intake: 44.5 μg/day and 0.56 μg/kg bw/day) [40] as well as Swedish participants that were selected with the specific aim of obtaining high intake variation (median AA intake: 45 μg/day) [28] had higher estimated AA intake than the general Swedish population (Figure 3 and Figure 4).

Generally, populations from Asia had lower estimated median/mean intakes compared to Europeans and Americans. Estimated intakes among Americans were slightly lower than those among Europeans (Figure 3, Figure 4, Figure 5 and Figure 6). Only one study used estimated dietary AA intake data from both Europe and the USA, and they showed higher median intake among Italian (28.2 μg/day) compared to American (up to 23.1 μg/day) pancreatic cancer cases and controls [41].

**Figure 7:**
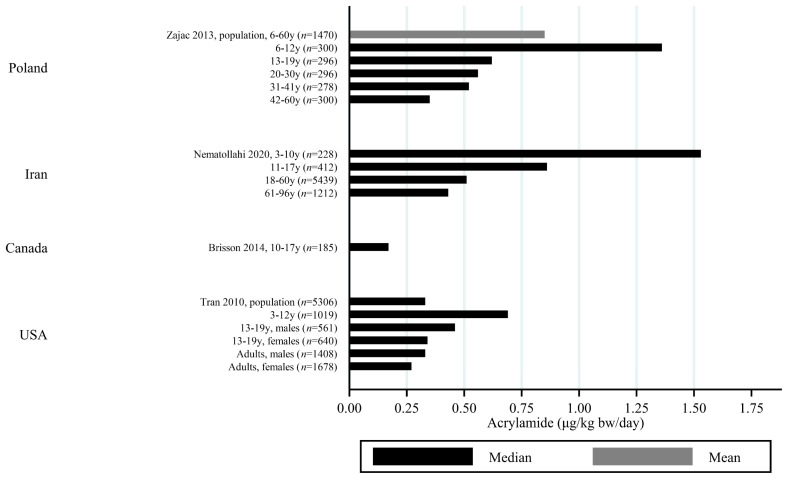
Median/mean estimated daily acrylamide intake per kg body weight (μg/kg bw/day), based on dietary questionnaire or both dietary questionnaire and dietary recall in studies including children by country.

**Figure 8:**
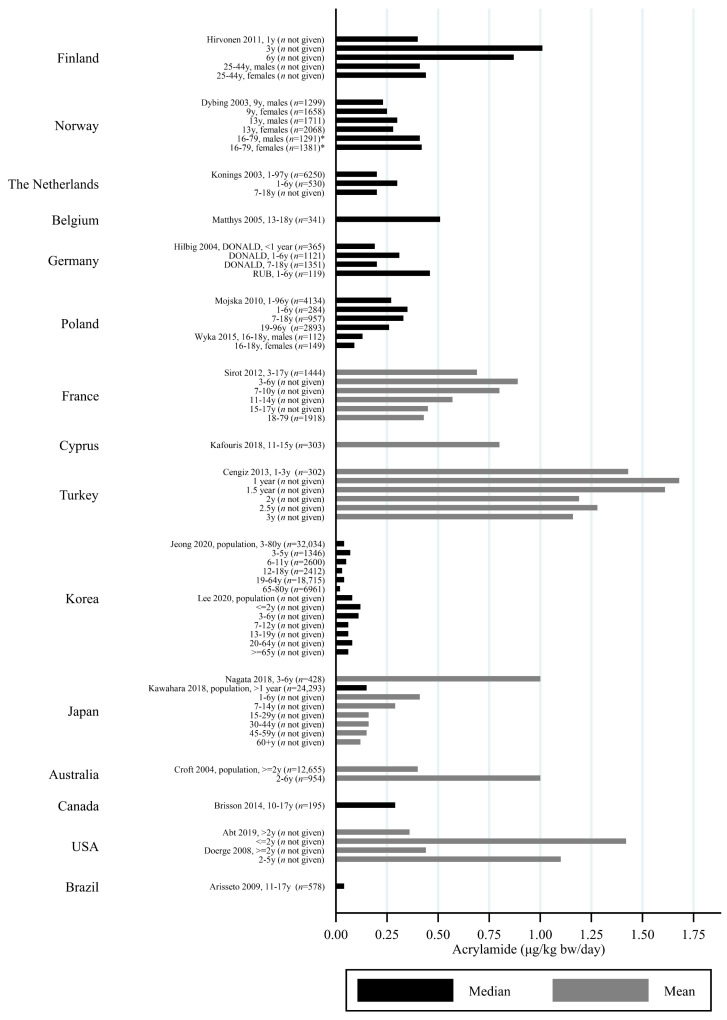
Median/mean estimated daily acrylamide intake per kg body weight (μg/kg bw/day) based on dietary recall in studies including children, by country. * Dybing et al., 2003 [43] estimated AA intake in adults using DQ.

All except one study on children estimated AA intake per kg bw. Children had estimated dietary AA intakes (per kg bw) that were up to three times higher than adults [30,42], and estimated dietary AA intake was generally inversely associated with age, so that elderlies had the lowest estimated intake and the youngest children had the highest estimated intake (Figure 7 and Figure 8). However, there were a few exceptions. For instance, one study reported that Norwegian children had lower estimated bw-adjusted dietary AA intake compared to adults [43] (Figure 8). Furthermore, the estimated dietary AA intake (per kg bw) among German infants (0.19 μg/kg bw/day) were lower than among 1–6-year olds (0.31 μg/kg bw/day) but comparable to the intake among 7–18 year olds (0.20 μg/kg bw/day) [44], and Finnish 1 year olds had lower estimated AA intakes (approximately 0.4 μg/kg bw/day) compared to 3 year olds (1.01 μg/kg bw/day) [45]. In contrast, Korean infants up to 2 years of age were found to have similar median AA intake (0.12 μg/kg bw/day) to the 3–6 year olds (0.11 μg/kg bw/day) [46], and Turkish 1 year olds had higher mean AA intake compared to 1.5–3 year olds [33]. A study from the USA also reported that infants below 2 years of age had higher mean AA intake (1.42 μg/kg bw/day) as compared to the rest of the population >2 years of age (0.36 μg/kg bw/day) [47] (Figure 8).

Only three studies provided estimated intakes that were not adjusted for bw. One of these studies reported that Polish 1–6 year olds had lower estimated AA intake (13.2 μg/day) than adults (23.1 μg/day), whereas 7–18 year olds had almost the same intake as adults (23.3 μg/day) [48]. An Australian study estimated that 2–6 year olds had slightly lower estimated AA intake (19 μg/day) than the total population (22 μg/day) [49], and the last study reported that Iranian 3–10 year olds had almost the same intake (43.9 μg/day) as 18–60 year olds (44.8 μg/day), whereas 11–17 year olds had higher estimated intakes than all other age groups (55.1 μg/day) [30].

### 3.3. Dietary Assessment Methods

DQs were used in 57 studies and DRs were used in 33 studies; one study used DQs for adults and DRs for children [43], six studies (five unique populations) compared the two methods, and four studies (three unique populations) combined the two methods. In the four studies that combined the two methods, one used a DQ, covering the past year, and derived information about portion size from a 24 h DR [50], whereas the other three studies did not describe the combination methods in detail [28,51,52]. In the five populations in which separate estimates of dietary AA intake based on DQ and DR were provided, two had higher estimates when using DQ compared to DR among adults (DQ vs. DQ median, P99: 0.48, 1.83 vs. 0.41, 1.36 μg/kg bw/day [26] and DQ vs. DR geometric mean, P10-P90: 24.7, 11.6–50.4 vs. 21.8, 7.1–59.5 μg/day [53]), two found lower estimates and less variance when using DQ compared to DR among adults (DQ vs. DR median, min-max: 0.11, 0.05–0.26 vs. 0.18, 0.06–0.71 μg/kg bw/day [54]) and adolescents (DQ vs. DR median, min–max: 0.17, 0.02–0.08 vs. 0.29, 0.00–5.78 μg/kg bw/day [39,55] and one found similar results using the two methods [27]. Most studies evaluated dietary habits based on single dietary assessments but a few used two repeated non-consecutive DRs [51,56] or DQs both before pregnancy and in the third trimester [24].

## 4. Discussion

We reviewed 101 studies, covering more than 1,238,703 individuals, including 27,598 children from 70 different populations, and found that the central estimates of dietary AA intake ranged from a median of 0.02 μg/kg bw/day among Korean 65–80 year olds [31] to a mean of 1.68 μg/kg bw/day among Turkish 1 year olds [33]. The lowest central estimate of dietary AA intake in our review is, thus, lower than the 0.1 μg/kg bw/day among Brazilian 11–17 year olds [57], reported as the lowest central estimate in 2011 by JECFA [17], and lower than the 0.4 μg/kg bw/day, which EFSA found among German adolescents and among adults from several European countries [5]. The highest central estimates of dietary AA intake in our review is also lower than the 2.0 μg/kg bw/day among children from the United Arab Emirates reported as the highest central estimate by JECFA in 2006 [10], and it is lower than the 1.9 μg/kg bw/day found among Belgian toddlers by EFSA [5]. The data on children from the United Arab Emirates and Belgium were not included in our review because, to our knowledge, these results have not been published in peer-reviewed papers. A number of countries have published data on dietary AA intake in national reports. Thus, the Norwegian Panel on Contaminants of the Norwegian Scientific Committee for Food Safety carried out an AA exposure assessment among 1635 1 year olds and found a mean (P95) intake of 0.9 (1.6) μg/kg bw/day [58], which is higher than previously found among older Norwegian children [43]. In a Danish report, children were found to have a mean (P95) estimated dietary AA intake of 0.21 (0.46) μg/kg bw/day while it was 0.39 (0.89) μg/kg bw/day among adults [59], which is, in contrast to most of the studies included in our review, illustrating higher estimated AA intake with lower age. Based on national data from New Zealand, the mean estimated dietary AA intake ranged from 0.72 μg/kg bw/day among women of 25 years or older to 2.22 μg/kg bw/day among 5–6 year olds, while toddlers had estimated intakes comparable to the 5–6 year olds (2.21 μg/kg bw/day), and infants had slightly lower estimated intakes (1.77 μg/kg bw/day) [9]. Thus, the New Zealand children had higher estimated intakes than any age group in any study population included in our review as well as in the reports by JECFA and EFSA [5,10,17].

### 4.1. Country-Specific Differences in AA Intake and Food Groups Contributing to the Total AA Intake

Based on the studies included in this review, European studies generally had higher estimated dietary intakes of AA than populations in other parts of the world, especially compared to studies from Asia that reported the lowest mean/median estimates of AA from diet, which might partly be explained by differences in study designs and methods used but might also reflect real differences in the dietary habits. Among Americans above the age of two, the main contributors to AA intake were breakfast cereal, French fries, potato chips, cookies, crackers and coffee [47]. In Swedish adults, the major sources of dietary AA were found to be crisp bread, coffee, fried potatoes, and other bread [40,60,61], and in Finnish adults it was coffee, starch-rich casseroles, rye bread and biscuits [45]. Likewise, coffee was the most important contributor to AA intake in women from the Netherlands, followed by Dutch spiced cake, French fries, potato crisps, and cookies [62], and in Italian and French adults, the main contributors were fried/baked potatoes/chips, coffee, biscuits, and bread [63,64]. Likewise, coffee, biscuits, and potatoes have been found to be main contributors of dietary AA among Japanese adults, but other vegetables, meat, and green tea were also important contributors [29,65], and in Chinese adults, vegetables and cereals accounted for the majority of the dietary AA intake [66]. It could be that the lower estimated AA intake among the Asian study populations compared to the European study populations reflect that these Asian populations consumed less coffee, potato crisps, soft breads and biscuits compared to other food products such as tea and raw foods. Furthermore, they might eat relatively more foods cooked in a way that results in lower AA formation, such as steaming instead of baking. However, due to differences in methods used to assess dietary intake, direct comparison between studies is uncertain. Thus, further studies using the same methods in populations with different food cultures are needed to compare dietary AA intakes across populations from different countries.

Most studies that report on the specific groups contributing to AA intake found that potato crisps, fried potatoes and other potato products only contribute to half or less than half of the total AA intake, and that other food items, such as breads and breakfast cereals, that are generally considered to be healthier, are also major contributors to AA intake [40,45,47,60,61,66]. These results illustrate that AA intake from the diet is not driven only by unhealthy foods, but also by foods that are considered as being healthy. Thus, AA intake can be associated with high fiber and essential micronutrient content, but also with ‘junk food’ and ‘highly processed fast food’ with high salt and fat content, as well as other unhealthy dietary patterns, such as low intake of healthy foods. Likewise, AA intake can be associated with other unhealthy lifestyle factors, low socio-economic status, and environmental stressors. Furthermore, factors associated with AA intake, such as consumption of fruits, vegetables, and garlic, alcohol intake, smoking, and low socioeconomic class may interact with AA intake/exposure, as well as providing protective as well as synergistic effects. The differences in food contributions within and across populations, e.g., the high crispbread contribution to total AA intake in Norway [26], make the evaluation of potential confounding and effect modification of any adverse health effects of AA by other dietary intake complex. This stresses the importance of conducting epidemiological studies of the potential adverse health effects of dietary exposure to AA, as experimental studies often rely on adding pure AA to water or food. Thus, the extrapolation of findings from experimental studies do not capture the complexity of human diet.

### 4.2. Age-Related Differences in AA Intake and Food Groups Contributing to the Total AA Intake

As JECFA [10,17] and EFSA [5], we found that young children were more exposed than adults, which is partly explained by their smaller body size and higher food intake compared to bw, and partly by their dietary preferences. Even in studies that did not adjust for bw, the estimated dietary intake among children and adolescents was similar to the intake among adults, indicating that children and adolescents consume more foods with high levels of AA. Though children likely consume, for instance, less coffee, which is often a main contributor to the total dietary AA exposure for adults, the consumption of, e.g., certain breakfast cereals and crackers may be more common in children than adults in some populations [67]. Bread, pastries, and potato products have been identified as the main contributors of AA among German children [44]. In Japanese children, the main dietary sources of AA were confectionaries, other snacks, vegetables, potatoes, starches, and cereal [32,68], and potatoes contributed more to the total AA intake in children aged 1–6 years (40%) compared to elderly (9%), whereas other vegetables contributed more among elderly (25%) compared to the children (15%) [32].

AA and other Maillard reaction compounds have also been detected in infant milk powder, as well as in breast milk, homemade baby food and commercial baby food, such as vegetable purée and crackers [44,69,70,71], and the EFSA found jarred baby food and follow-on formula to be the main contributors to total AA intake in infants [5]. Only one study in our review provided separate AA estimates for infants younger than 1 year and found lower estimated median dietary AA intake in German infants (0.19 μg/kg bw/day) compared to 1–6 year olds (0.31 μg/kg bw/day) [44]. Likewise, a French total diet study among non-breastfed infants aged 1–4, 5–6, 7–12, and 13–36 months found mean AA intakes between 0.51 (1–4 months) and 0.74 (13–36 months) μg/kg bw/day, when replacing values below LOD with the LOD and values below LOQ with the LOQ [72]. Thus, the French infants had lower estimated AA intakes than the French 3–10 year olds included in this review [73]. Furthermore, an Estonian study has estimated AA intake among infants from commercial baby foods alone and found mean AA intakes of 0.15 μg/kg bw/day among 4–5 month olds and 0.65 μg/kg bw/day among 6–11-month-old infants [71]. As most infants rely mainly on breastfeeding in the first months of life, their dietary intake of AA may be difficult to capture using DQs and DRs alone. However, a Swedish study measured AA directly in breastmilk and estimated that the mean AA intake among exclusively breastfed infants aged 0–6 months was 0.04 μg/kg bw/day, and the mean AA intake in the whole first year was 0.04–1.2 μg/kg bw/day, depending on the length of breast-feeding and the choice of baby food [69].

### 4.3. DQs Compared to DRs

In our review we did not observe systematic differences between AA estimates obtained based on DQs and DRs when we compared the estimates in the studies that relied on both methods. One study calculated no difference in estimated dietary AA intake [27], while most studies calculated lower [39,54,55], and the others higher [26,53] estimated dietary AA intake when using DQs compared to DRs. The EPIC study, which relied on 510 adults enrolled in different cohorts located in nine European countries, has reported a low correlation between AA intake calculated from DQs and a 24 h DR [53]. DQs are the most feasible methods to obtain detailed information on dietary habits over a long period for a large number of people. However, due to the length constrains of DQs, specific questions on portion sizes, preparation and cooking methods, as well as changes in intake over time are often left out. Thus, DQs are not typically designed to, and thus cannot effectively, estimate precise daily intakes of AA or any other foodborne contaminant at an individual level. On a population level, however, the DQs can be used to rank the participants into differential levels of intake. Another potential limitation, related to DQs covering a long period of time, or asking about dietary habits earlier in life, is the potential for recall bias and the impaired recollection of actual dietary habits, e.g., it has previously been found that overweight individuals are more likely to under-report their energy intake [74,75]. In contrast, a limitation of DRs is that they often cover only short-term intake and thus do not capture dietary habits over long periods. Furthermore, it is very labor intense for both participants and researchers to process the data if the participants are asked to consecutively report when, and how much, they eat and drink for several days and how the food was cooked, at which temperature and for how long, etc. However, a Finnish study on children and adults did take food preparation into account [45], which helps improve precision to some extent.

### 4.4. Limitations of DQs and DRs for Studies Estimating Total Dietary AA Intake

Most studies estimating dietary AA intake used data on the individual consumption of foods and drinks that were not specifically collected with the purpose of estimating AA, but for general purposes of the collection of overall dietary habits (Supplementary Table S1). A few studies used photographs or picture books to estimate portion sizes [24,28,35,42,48,73,76,77,78,79], while most studies had to rely on assumptions on standard portion sizes for the calculation of individual daily consumption. Likewise, most studies used standard assumptions about composition of the foods. For home-cooked mixed meals, the composition of foods might considerably vary, and thus the exact amounts of each component, e.g., flour or potatoes are difficult to estimate based on standard DQs and DRs [80]. Furthermore, when using DQs and DRs to estimate the dietary intake of AA, the estimates will depend on the assumptions of AA concentrations in the foods and drinks consumed. These concentrations vary across countries and regions [5], and the use of local food monitoring data, as compared to international data, will thus provide more precise estimates of the dietary intake of AA. Most of the studies included in this review relied on AA concentrations, measured nationally or even regionally [23,24,63,73], but some studies used AA estimates in food, based on international data [36,53,64,76,81,82,83,84,85,86,87]. Many studies have used AA levels in food from previous research or national reports, while others conducted their own AA analyses of food items using LC/MS [73], LC/MS/MS [46,47,57,66], HPLC/MS/MS [31], UPLC/MS/MS [88], GC/MS [30,33], or GC/MS/MS [48]. GC/MS and LC/MS show good agreement and are both accurate in measuring AA [89]. However, even when using appropriate analytical methods and measuring AA in local foods, the AA concentrations may vary within the same kinds of foods. For instance, the AA concentrations in dry coffee can vary from 0 to 1115 μg/kg, based on the composition of the coffee, as well as the roasting of the coffee beans, and the concentration in the consumed coffee also depends on the preparation of the coffee [90]. Likewise, according to European data compiled by EFSA, the concentration of AA in potato chips, also called French fries, varies from 14.5 to 1888 μg/kg for fried, fresh or pre-cooked, analyzed as sold [5], as the concentration of AA depends on concentrations of asparagine, fructose and other precursors, storage, and cooking methods. The variation in the actual frying temperature contributed most to the variation in AA concentrations, followed by the variation in actual frying time while no obvious effect of reducing sugars was found [91]. Similarly, for soft breads the AA concentration varies from 0 to 203 μg/kg before toasting [5], and in biscuits, crackers and crispbreads the AA concentration varies from 0 to 1600 μg/kg, again depending on composition, types of crops, storage and cooking methods [12,13,14], which adds imprecision to the use of DQs and DRs in assessing dietary AA intake, as detailed information on origin, brands and cooking methods are seldom available. Furthermore, the use of probabilistic methods, in which the concentration range within the same food items is utilized by modeling the intake based on the distribution of AA levels [92], can improve the validity of the dietary assessment methods. Other studies multiplied the consumption data with the median or mean levels of AA concentrations in foods and drinks. When conducting epidemiological studies, examining health effects or the disease burden of dietary intake of AA, it is recommended to also conduct sensitivity analyses using the 95th percentiles to identify dietary intake in a worst-case scenario.

Another limitation is that DQs and the software used to estimate consumption data may have grouped multiple foods that have different AA concentrations, and the DQs may also lack questions about some foods containing AA. For instance, AA can be detected in drinking water [93] and cow milk [94] and, although these concentrations are generally low, there could be foods and drinks that contribute to AA intake that are ignored.

Future studies of AA intake from diet could benefit from more detailed consumption data collected from DQs and DRs with a higher degree of details about food preparation methods, browning, and portion sizes. New methods of collecting data through smart phone or tablets, such as photos of foods, are encouraged to better capture variation in food preparation.

An additional limitation relates to the fact that most studies relied on the assessment of dietary habits only once, and variation over time has not been considered. A Norwegian study evaluated the AA intake of pregnant women and their children at 3 and 7 years of age but did not report the estimated AA intake among the children [22]. A recent Japanese study, evaluating the AA intake twice within one year using a combination of DQ and 12 days DR, reported that the ratios of intra- and inter-individual variation were 3.2 for men and 4.3 for women [95]. Preferably, future studies of adverse health outcomes that have a long latent time, such as cancer, would benefit from consumption data collection using DQs that covers long-term food habits that are assessed multiple times to evaluate intra-individual variation over time.

### 4.5. DQs and DRs Compared to Other Methods of Estimating Dietary AA Intake

Using DQs and DRs to obtain information on the individual consumption of different foods and drinks, combined with measured AA levels in local foods, is a cheaper method of estimating the total dietary intake of AA, as compared to measurements of AA concentrations in foods and drinks, as done in duplicate diet studies or analyses of biomarkers in urine or blood. In duplicate diet studies, AA is measured in duplicate portions of all foods and drinks consumed by the study participants. This method is more precise in estimating the dietary intake of AA, compared to DQs and DRs, but the use of duplicate diets is only feasible over a relatively short time period and, even so, it is much more expensive than DQs and DRs. Thus, duplicate diet studies are not practical when covering dietary habits over longer time periods in large study populations. In a Dutch study validating an AA database (*n* = 122), a very strong (*r* = 0.82) correlation was found between estimated dietary AA intake, as assessed by duplicate diet and DR, where both the duplicate diet and the DR covered 24 h [96]. Thus, DRs may be a good and cheaper alternative to analyses of duplicate diets for the estimation of AA intake. However, duplicate diet studies may be useful to estimate the precise intake of AA for a small number of individuals at an individual level. Such precise estimates are useful for linkage with biomarkers of early health effects to better understand the biological pathways underlying the toxicity of dietary AA intake and interactions with other dietary intakes, that can interact with the bio-activation and metabolism of AA, such as alcohol [97] and garlic [98,99].

For the evaluation of internal AA exposure, the urinary analysis of biomarkers of AA and related metabolites can provide an alternative method to estimate AA from all sources, and when combined with analysis of biomarkers specific to exposure to tobacco smoke, as well as data on dietary habits, it is possible to estimate the AA intake from the diet. Urinary biomarkers has been used to provide a snapshot of exposure over the previous day from diet in a hand-full of studies with humans, and among Norwegian pregnant women (*n* = 119) weak positive correlations (*r* = 0.19–0.34) were found between the amount of AA excreted in urine as mercapturic acid metabolites and total AA intake, as assessed by DQ and DR, with the correlations being strongest after bw adjustment [26]. Furthermore, among employees at the Norwegian Institute of Public Health (*n* = 53), the urinary excretion of AA metabolites was weakly moderately correlated (*r* = 0.35–0.57) with the intake of aspartic acid, protein, starch and coffee, as assessed by a 24 h DR [78], but not correlated with the concentration of hemoglobin (Hb) adducts of AA [100].

Analyses of Hb adducts from AA can provide an indication of the internal dose of AA and related exposures accumulated in the blood during the last 4 months [101]. More than 70 studies have evaluated Hb adducts from AA in humans and the large number of studies demonstrates that it is feasible to integrate this biomarker in epidemiological studies [102]. The correlation between total dietary AA intake and Hb adducts from AA has been reported to be none-to-low in some studies [53,100,103,104], while moderate correlations up to 0.39 [21,28,40,52,105] have been reported in other studies. Individual food and drink groups that are rich in AA have also been positively associated with Hb adduct levels in almost all studies reporting on diet but, compared to exposure to tobacco smoking, the correlations were weaker and less consistent across studies [102]. In a study from Sweden, the dietary AA explained 13% of the variation of the Hb adducts from AA in non-smokers and 25% in smokers, while 32% was explained by diet and smoking [28]. In a National Health and Nutrition Examination Survey (NHANES) study from the USA, which included children, adolescents, adults and seniors, the estimated AA intake from DRs [106] and a combination of DRs and DQs [50] have been associated with higher Hb adduct levels from AA. In this population, 34% [106] and 46% [50] of the variability in the Hb adduct levels from AA was explained by the estimated AA intake and the covariates in the models [50,106]. In a study of Canadian teenagers, a total AA intake, estimated through a DQ covering the months before blood donation, was associated with higher Hb adducts from AA, while no association was observed for the intake of dietary AA based on the diet two days before [55].

In addition to being able to measure the individual exposure, uptake and absorption of AA through analyses of internal dose more accurately, the inter-individual differences of AA metabolism that may be attributed to the activity and/or genetic polymorphisms of metabolic enzymes in individuals with different race, gender, age, smoking, dietary and alcohol status are taken into account when biomarkers of AA are analyzed [107]. Ideally, to better evaluate variation in the biomarkers of the internal AA such as Hb adducts from AA related to diet, simultaneously measurement of Hb adducts specific to smoking, e.g., ethylene oxide [108], and comprehensive analyses of diet is recommended.

The within-individual variation in background Hb adducts levels of AA has shown a relatively good agreement (correlation coefficient of 0.8) for blood samples collected 1–3 years apart (*n* = 45) [105]. A Swedish study of Hb adducts from AA measured three times in blood collected every 8 months over 20 months in 13 non-smokers found that the Hb adducts differed more between individuals (six-fold) than within the samples from the same individuals (two-fold) [52]. The variation in AA intake between individuals was larger when estimated from DQs than from Hb adduct levels indicating that it is more difficult to rank individuals at group levels according to AA intake based on DQs alone. It is, therefore, recommended that results from analyses of Hb adducts are integrated in the assessment of AA intake derived from well-designed DQs and well-characterized AA concentrations in food.

Analyses of biomarkers are costly and not feasible for very large study populations. In contrast, DQs and DRs allow estimation of AA intake for large study populations, which is needed in epidemiological studies assessing small increases in risk. Such epidemiological studies are needed as the extrapolation of findings of adverse effects of AA observed in animal studies is uncertain.

### 4.6. Limitations to Our Review

In this review we included all peer-reviewed scientific publications indexed by PubMed, in which AA intake was assessed from individual consumption data through DQs or DRs. We have not systematically reviewed all data available from national reports and non-peer-reviewed publications, but we included estimates available online from reports written in English in the discussion.

It should be noted that the reviewed studies consist of study populations that are not fully representative of the general population. Many of the studies consisted of individuals that are eligible and willing to participate in cohort studies and, although most of the cohorts were population based, selection bias cannot be ruled out. Some studies involved hospital patients [40,64,81], pregnant women [21,24,25] elderlies [29,109,110,111], and some were limited to employees at certain institutions [78] or individuals selected due to a high intake of food that is rich in AA [28]. Hospitalized patients are likely to have less homemade food and a different consumption compared to at home without disease, and pregnant women may have a lower intake of for instance coffee as compared to women in general [26]. Cohort studies that require the participants to complete long questionnaires may have an underrepresentation of individuals with less resources, and these individuals may have a different diet and higher AA exposure. However, although selection bias could be an issue in epidemiological studies evaluating the associations between dietary AA intake and health effects, the exposure misclassification of AA intake from the diet is a much greater concern of bias.

The number of food and drink items and the levels of details in the dietary methods varied markedly from study to study. Some studies included AA values for meatballs [34,45,60,112], sausages [34], and peanut butter [62,113], while others did not take these into account. Furthermore, the individual consumption data used in the different studies covered periods from 1976 [114,115] to 2018 [30], and the central estimates and measures of variance reported were not the same across all studies. Some studies reported both means and medians and found that the two central estimates were fairly close [116], while others showed large differences between means and medians [48].

All of these differences across studies makes the comparisons uncertain, but despite the limitations, we found that the variation across studies within the same country was relatively limited (Figure 2, Figure 3, Figure 4 and Figure 5), and studies that did deviate were most often studies performed on highly selected groups.

### 4.7. Perspectives

In the past 20 years, following the discovery of AA formation in a range of commonly consumed foods and drinks more than 100 studies on estimated dietary intake of AA has been published. A similar impressive number of experimental studies, method development studies and risk assessments have been performed across the world and large amounts of resources are also being spent on monitoring AA in foods and drinks as well as taking actions towards reducing AA concentrations in foods and drinks and controlling that these mitigations are taken. Nevertheless, as for now, it remains unknown if the dietary intake of AA affects human health or not, and it is impossible to say what level of dietary AA intake can be deemed safe, if any, as the assessment of AA from diet is uncertain.

AA is neurotoxic in animals and in humans, following occupational exposures [117,118,119,120], and dietary AA intake has been associated with a mild cognitive decline over a four-year period in non-smoking Chinese, elderly men, but not in women [109]. Furthermore AA crosses the human placenta [121], and the maternal intake of AA during pregnancy has been associated with restriction of intrauterine growth indicated by low birth weight, small-for-gestational age, and reduced birth head circumference [21,23,25,122]. Adverse effects on reproduction, growth and development including neurodevelopmental effects have been observed after gestational AA exposure in rodents, but the adverse effects of AA from the diet on the developing brain has, to our knowledge, not yet been studied in humans [120,123]. To date, the majority of epidemiological studies have focused on AA intake during adulthood and the potential link with cancer [5]. Studies of exposure during prenatal life and early infanthood are sparse, and more data are needed to elucidate the dietary AA intake in these vulnerable groups. Studies of other health effects than cancer are also needed, and studies of the health effects of exposure to AA from diet during critical windows of development and growth, such as during early life and puberty, are encouraged. In order to identify the associations with health outcomes, exposure to AA during the critical windows for these outcomes needs to be studied, and future dietary AA studies should focus on covering critical windows of exposure and vulnerability, as well as a broader range of outcomes. There is, especially, an urgent need for further research to elucidate whether dietary AA intake during early life might impair neurodevelopment, adversely affect neuro-function postnatally, and cause poor mental health and other adverse health effects later in life in humans.

## 5. Conclusions

Well-designed DQs and DRs are valuable methods to obtain information on individual dietary habits of large populations and linkage with well characterized AA food monitoring data can enable estimation of dietary AA intake in large study populations. The results of our review of the peer-reviewed studies on AA from total diet illustrate that these methods are capable of detecting variation between individuals and populations. Study populations of young children have relatively higher AA intakes per kg bw compared to adolescents and adults. The results suggest that the AA intake of individuals from European countries is higher than those elsewhere; however, the estimation of the AA intake is uncertain and the methodological differences between the studies hinder the direct comparison of AA intake between studies. It is, therefore, recommended that results from analyses of Hb adducts and information about the specific dietary patterns of foods and drinks contributing to AA are integrated into the assessment of AA intake derived from well-designed DQs or DRs, and validated AA food monitoring data. Dietary questionnaires can be used for a large population and provide information about dietary factors that might interact with the AA exposure, while the more expensive Hb adducts could be used for a subgroup within the population to provide more precise information about the effective dose of AA.

In conclusion, this overview shows that AA exposure is widespread, and that children are generally more exposed than adults, relative to their bodyweight. However, the large variation in estimated AA intake, across and within populations, highlights the potential for reduced AA intake among the highest exposed individuals.

## Figures and Tables

**Table 1:** Search strategy.

Facet 1	Facet 2	Facet 3
Acrylamide	Humans	Diet record
Acrylamides	Women	Nutrition assessment
	Men	Surveys and questionnaires
	Children	Dietary exposure
	Adolescents	Diet
	Adults	Feeding Behavior
		“Acrylamide Intake” *

* All search words, except for “Acrylamide Intake”, were MeSH terms.

## Data Availability

All results in this review are based on data obtained from the original peer reviewed publications referenced in the manuscript.

## Supplementary Materials

The following are available online at https://www.mdpi.com/article/10.3390/toxics9070155/s1, Table S1: Characteristics of the individual studies that evaluates total dietary intake of acrylamide (AA).

## References

[1] Tareke E., Rydberg P., Karlsson P., Eriksson S., Törnqvist M. (2002). Analysis of Acrylamide, a Carcinogen Formed in Heated Foodstuffs. J. Agric. Food Chem..

[2] Mottram D.S., Wedzicha B.L., Dodson A.T. (2002). Acrylamide is formed in the Maillard reaction. Nat. Cell Biol..

[3] Stadler R.H., Blank I., Varga N., Robert F., Hau J., Guy P.A., Robert M.-C., Riediker S. (2002). Acrylamide from Maillard reaction products. Nat. Cell Biol..

[4] World Health Organization International Agency for Research on Cancer (1994). Some Industrial Chemicals. IARC Monograhs on the Evaluation of Carcinogenic Risks to Humans.

[5] EFSA (2015). Scientific Opinion on acrylamide in food. EFSA J..

[6] Government of Canada Sampling Plan for the First Phase of the Acrylamide Monitoring Program 2009 [updated August 2009]. https://www.canada.ca/en/health-canada/services/food-nutrition/food-safety/chemical-contaminants/food-processing-induced-chemicals/acrylamide/sampling-plan-first-phase-acrylamide-monitoring-program-chemical-contaminants.html.

[7] U.S. Food and Drug Administration Survey Data on Acrylamide in Food: Total Diet Study Results 2018 [updated 25 January 2018]. https://www.fda.gov/food/chemicals/survey-data-acrylamide-food-total-diet-study-results.

[8] Food Standards Australia New Zealand (2014). 24th Australian Total Diet Study.

[9] Cressey P., Thomson B., Ashworth M., Grounds P., McGill E. (2012). Acrylamide in New Zealand Food and Updated Exposure Assess-ment.

[10] FAO/WHO (2006). Safety Evaluation of Certain Contaminants in Food.

[11] Sörgel F., Weissenbacher R., Kinzig-Schippers M., Hofmann A., Illauer M., Skott A., Landersdorfer C. (2002). Acrylamide: Increased Concentrations in Homemade Food and First Evidence of Its Variable Absorption from Food, Variable Metabolism and Placental and Breast Milk Transfer in Humans. Chemotherapy.

[12] Elmore J.S., Koutsidis G., Dodson A.T., Mottram N.S., Wedzicha B.L. (2006). The Effect of Cooking on Acrylamide and Its Precursors in Potato, Wheat and Rye. Chemistry and Biology of Pteridines and Folates.

[13] Stadler R.H. (2005). Acrylamide Formation in Different Foods and Potential Strategies for Reduction.

[14] Ishihara K., Matsunaga A., Miyoshi T., Nakamura K., Nakayama T., Ito S., Koga H. (2005). Formation of Acrylamide in a Processed Food Model System, and Examination of Inhibitory Conditions. J. Food Hyg. Soc. Jpn..

[15] Pedreschi F., Mariotti M.S., Granby K. (2013). Current issues in dietary acrylamide: Formation, mitigation and risk assessment. J. Sci. Food Agric..

[16] Lachenmeier D.W., Schwarz S., Teipel J., Hegmanns M., Kuballa T., Walch S.G., Breitling-Utzmann C.M. (2018). Potential Antagonistic Effects of Acrylamide Mitigation during Coffee Roasting on Furfuryl Alcohol, Furan and 5-Hydroxymethylfurfural. Toxics.

[17] FAO/WHO (2011). Safety Evaluation of Certain Contaminants in Food.

[18] Pelucchi C., Bosetti C., Galeone C., La Vecchia C. (2015). Dietary acrylamide and cancer risk: An updated meta-analysis. Int. J. Cancer.

[19] Riboldi B.P., Vinhas Álvaro M., Moreira J.D. (2014). Risks of dietary acrylamide exposure: A systematic review. Food Chem..

[20] Virk-Baker M.K., Nagy T.R., Barnes S., Groopman J. (2014). Dietary acrylamide and human cancer: A systematic review of literature. Nutr. Cancer.

[21] Duarte-Salles T., Von Stedingk H., Granum B., Gützkow K.B., Rydberg P., Törnqvist M., Mendez M.A., Brunborg G., Brantsæter A.L., Meltzer H.M. (2013). Dietary Acrylamide Intake during Pregnancy and Fetal Growth—Results from the Norwegian Mother and Child Cohort Study (MoBa). Environ. Health Perspect..

[22] Kadawathagedara M., Botton J., De Lauzon-Guillain B., Meltzer H.M., Alexander J., Brantsaeter A.L., Haugen M., Papadopoulou E. (2018). Dietary acrylamide intake during pregnancy and postnatal growth and obesity: Results from the Norwegian Mother and Child Cohort Study (MoBa). Environ. Int..

[23] Kadawathagedara M., Tong A.C.H., Heude B., Forhan A., Charles M.A., Sirot V., Botton J. (2016). The EDEN mother-child cohort study group Dietary acrylamide intake during pregnancy and anthropometry at birth in the French EDEN mother-child cohort study. Environ. Res..

[24] Chan-Hon-Tong A., Charles M.A., Forhan A., Heude B., Sirot V. (2013). Exposure to food contaminants during pregnancy. Sci. Total. Environ..

[25] Nagata C., Konishi K., Wada K., Tamura T., Goto Y., Koda S., Mizuta F., Iwasa S. (2018). Maternal Acrylamide Intake during Pregnancy and Sex Hormone Levels in Maternal and Umbilical Cord Blood and Birth Size of Offspring. Nutr. Cancer.

[26] Brantsæter A.L., Haugen M., De Mul A., Bjellaas T., Becher G., Van Klaveren J., Alexander J., Meltzer H.M. (2008). Exploration of different methods to assess dietary acrylamide exposure in pregnant women participating in the Norwegian Mother and Child Cohort Study (MoBa). Food Chem. Toxicol..

[27] Kotemori A., Ishihara J., Nakadate M., Sawada N., Iwasaki M., Sobue T., Tsugane S. (2018). Validity of a Self-administered Food Frequency Questionnaire for the Estimation of Acrylamide Intake in the Japanese Population: The JPHC FFQ Validation Study. J. Epidemiol..

[28] Wirfält E., Paulsson B., Törnqvist M., Axmon A., Hagmar L., Wirf E. (2007). Associations between estimated acrylamide intakes, and hemoglobin AA adducts in a sample from the Malmö Diet and Cancer cohort. Eur. J. Clin. Nutr..

[29] Liu R., Sobue T., Kitamura T., Kitamura Y., Ishihara J., Kotemori A., Zha L., Ikeda S., Sawada N., Iwasaki M. (2019). Dietary Acrylamide Intake and Risk of Esophageal, Gastric, and Colorectal Cancer: The Japan Public Health Center-based Prospective Study. Cancer Epidemiol. Biomark. Prev..

[30] Nematollahi A., Kamankesh M., Hosseini H., Ghasemi J., Hosseini-Esfahani F., Mohammadi A., Khaneghah A.M. (2020). Acrylamide content of collected food products from Tehran’s market: A risk assessment study. Environ. Sci. Pollut. Res..

[31] Jeong H., Hwang S., Kwon H. (2020). Survey for acrylamide in processed foods from Korean market and individual exposure estimation using a non-parametric probabilistic model. Food Addit. Contam. Part. A.

[32] Kawahara J., Imaizumi Y., Kuroda K., Aoki Y., Suzuki N. (2018). Estimation of long-term dietary exposure to acrylamide of the Japanese people. Food Addit. Contam. Part. A.

[33] Cengiz M.F., Gündüz C.P.B. (2013). Acrylamide exposure among Turkish toddlers from selected cereal-based baby food samples. Food Chem. Toxicol..

[34] Svensson K., Abramsson L., Becker W., Glynn A., Hellenäs K.-E., Lind Y., Rosén J. (2003). Dietary intake of acrylamide in Sweden. Food Chem. Toxicol..

[35] Mojska H., Gielecińska I., Zielińska A., Winiarek J., Sawicki W. (2015). Estimation of exposure to dietary acrylamide based on mercapturic acids level in urine of Polish women post partum and an assessment of health risk. J. Expo. Sci. Environ. Epidemiol..

[36] Lujan-Barroso L., González C.A., Slimani N., Obón-Santacana M., Ferrari P., Freisling H., Overvad K., Clavel-Chapelon F., Boutron-Ruault M.-C., Racine A. (2014). Dietary intake of acrylamide and esophageal cancer risk in the European Prospective Investigation into Cancer and Nutrition cohort. Cancer Causes Control..

[37] Mucci L.A., Sandin S., Bälter K., Adami H.-O., Magnusson C., Weiderpass E. (2005). Acrylamide Intake and Breast Cancer Risk in Swedish Women. JAMA.

[38] Kotemori A., Ishihara J., Zha L., Liu R., Sawada N., Iwasaki M., Sobue T., Tsugane S. (2017). The JPHC Study Group Dietary acrylamide intake and risk of breast cancer: The Japan Public Health Center-based Prospective Study. Cancer Sci..

[39] Normandin L., Bouchard M., Ayotte P., Blanchet C., Becalski A., Bonvalot Y., Phaneuf D., Lapointe C., Gagné M., Courteau M. (2013). Dietary exposure to acrylamide in adolescents from a Canadian urban center. Food Chem. Toxicol..

[40] Wilson K.M., Bälter K., Adami H.-O., Grönberg H., Vikström A.C., Paulsson B., Törnqvist M., Mucci L.A. (2009). Acrylamide exposure measured by food frequency questionnaire and hemoglobin adduct levels and prostate cancer risk in the Cancer of the Prostate in Sweden Study. Int. J. Cancer.

[41] Pelucchi C., Rosato V., Bracci P.M., Li D., Neale R.E., Lucenteforte E., Serraino D., Anderson K.E., Fontham E., Holly E.A. (2017). Dietary acrylamide and the risk of pancreatic cancer in the International Pancreatic Cancer Case–Control Consortium (PanC4). Ann. Oncol..

[42] Zając J., Bojar I., Helbin J., Kolarzyk E., Potocki A., Strzemecka J., Owoc A. (2013). Dietary acrylamide exposure in chosen population of South Poland. Ann. Agric. Environ. Med..

[43] Dybing E. (2003). Risk Assessment of Acrylamide in Foods. Toxicol. Sci..

[44] Hilbig A., Freidank N., Kersting M., Wilhelm M., Wittsiepe J. (2004). Estimation of the dietary intake of acrylamide by German infants, children and adolescents as calculated from dietary records and available data on acrylamide levels in food groups. Int. J. Hyg. Environ. Health.

[45] Hirvonen T., Jestoi M., Tapanainen H., Valsta L., Virtanen S.M., Sinkko H., Kronberg-Kippilä C., Kontto J., Virtamo J., Simell O. (2011). Dietary acrylamide exposure among Finnish adults and children: The potential effect of reduction measures. Food Addit. Contam. Part. A.

[46] Lee S., Kim H.J. (2020). Dietary Exposure to Acrylamide and Associated Health Risks for the Korean Population. Int. J. Environ. Res. Public Health.

[47] Abt E., Robin L.P., McGrath S., Srinivasan J., DiNovi M., Adachi Y., Chirtel S. (2019). Acrylamide levels and dietary exposure from foods in the United States, an update based on 2011-2015 data. Food Addit. Contam. Part. A.

[48] Mojska H., Gielecińska I., Szponar L., Ołtarzewski M. (2010). Estimation of the dietary acrylamide exposure of the Polish population. Food Chem. Toxicol..

[49] Tong P., Fuentes D., Hambridge T. (2004). Australian survey of acrylamide in carbohydrate-based foods. Food Addit. Contam..

[50] Tran N., Barraj L., Murphy M., Bi X. (2010). Dietary Acrylamide Exposure and Hemoglobin Adducts—National Health and Nutrition Examination Survey (2003–04). Food Chem. Toxicol..

[51] Claeys W.L., Baert K., Mestdagh F., Vercammen J., Daenens P., De Meulenaer B., Maghuin-Rogister G., Huyghebaert A. (2010). Assessment of the acrylamide intake of the Belgian population and the effect of mitigation strategies. Food Addit. Contam. Part. A.

[52] Vikström A.C., Warholm M., Paulsson B., Axmon A., Wirfält E., Törnqvist M. (2012). Hemoglobin adducts as a measure of variations in exposure to acrylamide in food and comparison to questionnaire data. Food Chem. Toxicol..

[53] Ferrari P., Freisling H., Duell E.J., Kaaks R., Lujan-Barroso L., Clavel-Chapelon F., Boutron-Ruault M.-C., Nailler L., Polidoro S., Mattiello A. (2012). Challenges in estimating the validity of dietary acrylamide measurements. Eur. J. Nutr..

[54] Yamamoto J., Ishihara J., Kotemori A., Nakadate M., Sobue T. (2018). Validity of Estimated Acrylamide Intake by the Dietary Record Method and Food Frequency Questionnaire in Comparison with a Duplicate Method: A Pilot Study. J. Nutr. Sci. Vitaminol..

[55] Brisson B., Ayotte P., Normandin L., Gaudreau E., Bienvenu J.-F., Fennell T.R., Blanchet C., Phaneuf D., Lapointe C., Bonvalot Y. (2013). Relation between dietary acrylamide exposure and biomarkers of internal dose in Canadian teenagers. J. Expo. Sci. Environ. Epidemiol..

[56] Wong W.W., Chung S.W., Lam C.-H., Ho Y., Xiao Y. (2014). Dietary exposure of Hong Kong adults to acrylamide: Results of the first Hong Kong Total Diet Study. Food Addit. Contam. Part. A.

[57] Arisseto A.P.T., de Figueiredo M.C., Govaert Y., van Loco J., Fraselle S., Degroodt J.-M., Rosseto Caroba D.C. (2009). Contribution of selected foods to acrylamide intake by a population of Brazilian adolescents. Food Sci. Technol..

[58] Brantsæter A.L., Knutsen H.K., Lillegaard I.T.L., Amlund H., Eriksen G.S., Fæste C.K., Kvalem H.E., Miles C.O., Oskam I., Ruus A. (2018). Risk Assessment of Dietary Exposure to Acrylamide in the Norwegian Population. Eur. J. Nutr. Food Saf..

[59] Petersen A., Fromberg A., Andersen J.H., Sloth J.J., Granby K., Duedahl-Olesen L., Rasmussen P.H., Fagt S., Cederberg T.L., Christensenet T. (2013). Chemical Contaminants. Food Monitoring 2004–2011.

[60] Mucci L.A., Adami H.-O., Wolk A. (2005). Prospective study of dietary acrylamide and risk of colorectal cancer among women. Int. J. Cancer.

[61] Mucci L.A., Lindblad P., Steineck G., Adami H.-O. (2004). Dietary acrylamide and risk of renal cell cancer. Int. J. Cancer.

[62] Hogervorst J.G., Schouten L., Konings E.J., Goldbohm R.A., Brandt P.V.D. (2007). A Prospective Study of Dietary Acrylamide Intake and the Risk of Endometrial, Ovarian, and Breast Cancer. Cancer Epidemiol. Biomark. Prev..

[63] Mancini F.R., Sirot V., Busani L., Volatier J.-L., Hulin M. (2015). Use and impact of usual intake models on dietary exposure estimate and risk assessment of chemical substances: A practical example for cadmium, acrylamide and sulphites. Food Addit. Contam. Part. A.

[64] Pelucchi C., Galeone C., Talamini R., Negri E., Polesel J., Serraino D., La Vecchia C. (2011). Dietary acrylamide and pancreatic cancer risk in an Italian case–control study. Ann. Oncol..

[65] Nagata C., Konishi K., Tamura T., Wada K., Tsuji M., Hayashi M., Takeda N., Yasuda K. (2015). Associations of Acrylamide Intake with Circulating Levels of Sex Hormones and Prolactin in Premenopausal Japanese Women. Cancer Epidemiol. Biomark. Prev..

[66] Gao J., Zhao Y., Zhu F., Ma Y., Li X., Miao H., Wu Y. (2016). Dietary exposure of acrylamide from the fifth Chinese Total Diet Study. Food Chem. Toxicol..

[67] Pedersen A.N., Christensen T., Matthiessen J., Knudsen V.K., Rosenlund-Sørensen M., Biltoft-Jensen A., Hinsch H.J., Ygil K.H., Kørup K., Saxholt E. (2015). Dietary Habits in Denmark 2011–2013.

[68] (2017). Acrylamide Intake with Urinary Sex Hormone Levels among Preschool Japanese Children. Am. J. Epidemiol..

[69] Fohgelberg P., Rosén J., Hellenäs K.-E., Abramsson-Zetterberg L. (2005). The acrylamide intake via some common baby food for children in Sweden during their first year of life—an improved method for analysis of acrylamide. Food Chem. Toxicol..

[70] Petrarca M.H., Rosa M.A., Queiroz S.C.N., Godoy H.T. (2017). Simultaneous determination of acrylamide and 4-hydroxy-2,5-dimethyl-3(2 H )-furanone in baby food by liquid chromatography–tandem mass spectrometry. J. Chromatogr. A.

[71] Elias A., Roasto M., Reinik M., Nelis K., Nurk E., Elias T. (2017). Acrylamide in commercial foods and intake by infants in Estonia. Food Addit. Contam. Part. A.

[72] Sirot V., Rivière G., Leconte S., Vin K., Traore T., Jean J., Carne G., Gorecki S., Veyrand B., Marchand P. (2019). French infant total diet study: Dietary exposure to heat-induced compounds (acrylamide, furan and polycyclic aromatic hydrocarbons) and associated health risks. Food Chem. Toxicol..

[73] Sirot V., Hommet F., Tard A., Leblanc J.-C. (2012). Dietary acrylamide exposure of the French population: Results of the second French Total Diet Study. Food Chem. Toxicol..

[74] Previdelli A.N., Gómez G., Kovalskys I., Fisberg M., Cortés L.Y., Pareja R.G., Liria M.R., García M.C.Y., Herrera-Cuenca M., Rigotti A. (2019). Prevalence and determinants of misreporting of energy intake among Latin American populations: Results from ELANS study. Nutr. Res..

[75] Tam K.W., Veerman J.L. (2019). Prevalence and characteristics of energy intake under-reporting among Australian adults in 1995 and 2011 to 2012. Nutr. Diet..

[76] Freisling H., Moskal A., Ferrari P., Nicolas G., Knaze V., Clavel-Chapelon F., Boutron-Ruault M.-C., Nailler L., Teucher B., Grote V.A. (2012). Dietary acrylamide intake of adults in the European Prospective Investigation into Cancer and Nutrition differs greatly according to geographical region. Eur. J. Nutr..

[77] Hirvonen T.P., Kontto J., Jestoi M., Valsta L.M., Peltonen K., Pietinen P., Virtanen S.M., Sinkko H., Kronbergkippila C., Albanes D. (2010). Dietary acrylamide intake and the risk of cancer among Finnish male smokers. Cancer Causes Control..

[78] Bjellaas T., Stølen L.H., Haugen M., Paulsen J.E., Alexander J., Lundanes E., Becher G. (2007). Urinary acrylamide metabolites as biomarkers for short-term dietary exposure to acrylamide. Food Chem. Toxicol..

[79] Wyka J., Tajner-Czopek A., Broniecka A., Piotrowska E., Bronkowska M., Biernat J. (2015). Estimation of dietary exposure to acrylamide of Polish teenagers from an urban environment. Food Chem. Toxicol..

[80] Dybing E., Farmer P., Andersen M., Fennell T., Lalljie S., Müller D., Olin S., Petersen B., Schlatter J., Scholz G. (2005). Human exposure and internal dose assessments of acrylamide in food. Food Chem. Toxicol..

[81] Pelucchi C., Galeone C., Maso L.D., Talamini R., Montella M., Ramazzotti V., Negri E., Franceschi S., La Vecchia C. (2006). Dietary acrylamide and renal cell cancer. Int. J. Cancer.

[82] Pelucchi C., Galeone C., Levi F., Negri E., Franceschi S., Talamini R., Bosetti C., Giacosa A., La Vecchia C. (2006). Dietary acrylamide and human cancer. Int. J. Cancer.

[83] Pelucchi C., Galeone C., Negri E., Bosetti C., Serraino D., Montella M., Talamini R., La Vecchia C. (2016). Dietary acrylamide and the risk of endometrial cancer: An Italian case-control study. Nutr. Cancer.

[84] Obón-Santacana M., Kaaks R., Slimani N., Lujan-Barroso L., Freisling H., Ferrari P., Dossus L., Chabbert-Buffet N., Baglietto L., Fortner R. (2014). Dietary intake of acrylamide and endometrial cancer risk in the European Prospective Investigation into Cancer and Nutrition cohort. Br. J. Cancer.

[85] Obón-Santacana M., Lujan-Barroso L., Freisling H., Cadeau C., Fagherazzi G., Boutron-Ruault M.-C., Kaaks R., Fortner R., Boeing H., Quirós J.R. (2016). Dietary and lifestyle determinants of acrylamide and glycidamide hemoglobin adducts in non-smoking postmenopausal women from the EPIC cohort. Eur. J. Nutr..

[86] Obón-Santacana M., Peeters P.H., Freisling H., Dossus L., Clavel-Chapelon F., Baglietto L., Schock H., Fortner R., Boeing H., Tjonneland A. (2015). Dietary Intake of Acrylamide and Epithelial Ovarian Cancer Risk in the European Prospective Investigation into Cancer and Nutrition (EPIC) Cohort. Cancer Epidemiol. Biomark. Prev..

[87] Obón-Santacana M., Slimani N., Lujan-Barroso L., Travier N., Hallmans G., Freisling H., Ferrari P., Boutron-Ruault M.C., Racine A., Clavel F. (2013). Dietary intake of acrylamide and pancreatic cancer risk in the European Prospective Investigation into Cancer and Nutrition (EPIC) cohort. Ann. Oncol..

[88] Kafouris D., Stavroulakis G., Christofidou M., Iakovou X., Christou E., Paikousis L., Christodoulidou M., Ioannou-Kakouri E., Yiannopoulos S. (2018). Determination of acrylamide in food using a UPLC–MS/MS method: Results of the official control and dietary exposure assessment in Cyprus. Food Addit. Contam. Part. A.

[89] Castle L., Eriksson S. (2005). Analytical methods used to measure acrylamide concentrations in foods. J. AOAC Int..

[90] Schouten M.A., Tappi S., Romani S. (2020). Acrylamide in coffee: Formation and possible mitigation strategies–A review. Crit. Rev. Food Sci. Nutr..

[91] Sanny M., Jinap S., Bakker E., van Boekel M., Luning P. (2012). Possible causes of variation in acrylamide concentration in French fries prepared in food service establishments: An observational study. Food Chem..

[92] Mills C., Tlustos C., Evans R., Matthews W. (2008). Dietary Acrylamide Exposure Estimates for the United Kingdom and Ireland: Comparison between Semiprobabilistic and Probabilistic Exposure Models. J. Agric. Food Chem..

[93] World Health Organization (WHO) (2011). Acrylamide in Drinking-Water. Background Document for Development of WHO Guidelines for Drinking-Water Quality.

[94] Pabst K., Mathar W., Palavinskas R., Meisel H., Blüthgen A., Klaffke H. (2005). Acrylamide–occurrence in mixed concentrate feed for dairy cows and carry-over into milk. Food Addit. Contam..

[95] Kito K., Ishihara J., Yamamoto J., Hosoda T., Kotemori A., Takachi R., Nakamura K., Tanaka J., Yamaji T., Shimazu T. (2020). Variations in the estimated intake of acrylamide from food in the Japanese population. Nutr. J..

[96] Konings E.J.M., Hogervorst J.G.F., Van Rooij L., Schouten L., Sizoo E.A., Van Egmond H.P., Goldbohm R.A., Brandt P.V.D. (2010). Validation of a database on acrylamide for use in epidemiological studies. Eur. J. Clin. Nutr..

[97] Vikström A.C., Wilson K.M., Paulsson B., Athanassiadis I., Grönberg H., Adami H.-O., Adolfsson J., Mucci L.A., Bälter K., Törnqvist M. (2010). Alcohol influence on acrylamide to glycidamide metabolism assessed with hemoglobin-adducts and questionnaire data. Food Chem. Toxicol..

[98] Li J., Zuo J., Qiao X., Zhang Y., Xu Z. (2016). Effect of garlic powder on acrylamide formation in a low-moisture model system and bread baking. J. Sci. Food Agric..

[99] Taubert D., Glöckner R., Müller D., Schömig E. (2006). The garlic ingredient diallyl sulfide inhibits cytochrome P450 2E1 dependent bioactivation of acrylamide to glycidamide. Toxicol. Lett..

[100] Bjellaas T., Olesen P.T., Frandsen H., Haugen M., Stølen L.H., Paulsen J.E., Alexander J., Lundanes E., Becher G. (2007). Comparison of Estimated Dietary Intake of Acrylamide with Hemoglobin Adducts of Acrylamide and Glycidamide. Toxicol. Sci..

[101] Törnqvist M. (2006). Acrylamide in Food: The Discovery and Its Implications. Chem. Biol. Pteridines Folates.

[102] Pedersen M., Vryonidis E., Joensen A., Halldorsson T.I., Olsen S.F., Törnqvist M. Hemoglobin adducts of acrylamide in human blood—What has been done and what is next?.

[103] Kütting B., Schettgen T., Beckmann M.W., Angerer J., Drexler H. (2005). Influence of Diet on Exposure to Acrylamide—Reflections on the Validity of a Questionnaire. Ann. Nutr. Metab..

[104] Kütting B., Uter W., Drexler H. (2007). The association between self-reported acrylamide intake and hemoglobin adducts as biomarkers of exposure. Cancer Causes Control..

[105] Wilson K.M., Vesper H.W., Tocco P., Sampson L., Rosén J., Hellenäs K.-E., Törnqvist M., Willett W.C. (2009). Validation of a food frequency questionnaire measurement of dietary acrylamide intake using hemoglobin adducts of acrylamide and glycidamide. Cancer Causes Control..

[106] Duke T.J., Ruestow P.S., Marsh G.M. (2017). The influence of demographic, physical, behavioral, and dietary factors on hemoglobin adduct levels of acrylamide and glycidamide in the general U.S. population. Crit. Rev. Food Sci. Nutr..

[107] Li D., Wang P., Liu Y., Hu X., Chen F. (2016). Metabolism of Acrylamide: Interindividual and Interspecies Differences as Well as the Application as Biomarkers. Curr. Drug Metab..

[108] Von Stedingk H., Vikström A.C., Rydberg P., Pedersen M., Nielsen J.K.S., Segerbäck D., Knudsen L.E., Törnqvist M. (2011). Analysis of Hemoglobin Adducts from Acrylamide, Glycidamide, and Ethylene Oxide in Paired Mother/Cord Blood Samples from Denmark. Chem. Res. Toxicol..

[109] Liu Z.-M., Tse L.A., Chen B., Wu S., Chan D., Kowk T., Woo J., Xiang Y.-T., Wong S.Y.-S. (2017). Dietary acrylamide exposure was associated with mild cognition decline among non-smoking Chinese elderly men. Sci. Rep..

[110] Liu Z.-M., Tse L.A., Ho S.C., Wu S., Chen B., Chan D., Wong S.Y.-S. (2017). Dietary acrylamide exposure was associated with increased cancer mortality in Chinese elderly men and women: A 11-year prospective study of Mr. and Ms. OS Hong Kong. J. Cancer Res. Clin. Oncol..

[111] McCullough M.L., Hodge R.A., Um C.Y., Gapstur S.M. (2019). Dietary Acrylamide Is Not Associated with Renal Cell Cancer Risk in the CPS-II Nutrition Cohort. Cancer Epidemiol. Biomark. Prev..

[112] Mucci L.A., Dickman P., Steineck G., Adami H.-O., Augustsson K. (2003). Dietary acrylamide and cancer of the large bowel, kidney, and bladder: Absence of an association in a population-based study in Sweden. Br. J. Cancer.

[113] Hogervorst J.G., Schouten L., Konings E.J., Goldbohm R.A., Brandt P.V.D. (2008). Dietary acrylamide intake and the risk of renal cell, bladder, and prostate cancer. Am. J. Clin. Nutr..

[114] Hogervorst J.G., Fortner R., Mucci L.A., Tworoger S., Eliassen A.H., Hankinson S.E., Wilson K.M. (2013). Associations between Dietary Acrylamide Intake and Plasma Sex Hormone Levels. Cancer Epidemiol. Biomark. Prev..

[115] Wilson K.M., Mucci L.A., Rosner B.A., Willett W.C. (2010). A Prospective Study on Dietary Acrylamide Intake and the Risk for Breast, Endometrial, and Ovarian Cancers. Cancer Epidemiol. Biomark. Prev..

[116] Larsson S.C., Åkesson A., Bergkvist L., Wolk A. (2009). Dietary acrylamide intake and risk of colorectal cancer in a prospective cohort of men. Eur. J. Cancer.

[117] Calleman C., Wu Y., He F., Tian G., Bergmark E., Zhang S., Deng H., Wang Y., Crofton K., Fennell T. (1994). Relationships between Biomarkers of Exposure and Neurological Effects in a Group of Workers Exposed to Acrylamide. Toxicol. Appl. Pharmacol..

[118] Hagmar L., Törnqvist M., Nordander C., Rosén I., Bruze M., Kautiainen A., Magnusson A.-L., Malmberg B., Aprea P., Granath F. (2001). Health effects of occupational exposure to acrylamide using hemoglobin adducts as biomarkers of internal dose. Scand. J. Work. Environ. Health.

[119] Pennisi M., Malaguarnera G., Puglisi V., Vinciguerra L., Vacante M., Malaguarnera M. (2013). Neurotoxicity of Acrylamide in Exposed Workers. Int. J. Environ. Res. Public Health.

[120] Lindeman B., Johansson Y., Andreassen M., Husøy T., Dirven H., Hofer T., Knutsen H.K., Caspersen I.H., Vejrup K., Paulsen R.E. (2021). Does the food processing contaminant acrylamide cause developmental neurotoxicity? A review and identification of knowledge gaps. Reprod. Toxicol..

[121] Schettgen T., Hornig M., Beckmann M.W., Weiss T., Drexler H., Angerer J. (2004). Trans-placental exposure of neonates to acrylamide? A pilot study. Int. Arch. Occup. Environ. Health.

[122] Pedersen M., Von Stedingk H., Botsivali M., Agramunt S., Alexander J., Brunborg G., Chatzi L., Fleming S.J., Fthenou E., Granum B. (2012). Birth Weight, Head Circumference, and Prenatal Exposure to Acrylamide from Maternal Diet: The European Prospective Mother–Child Study (NewGeneris). Environ. Health Perspect..

[123] Matoso V., Bargi-Souza P., Ivanski F., Romano M.A., Romano R.M. (2019). Acrylamide: A review about its toxic effects in the light of Developmental Origin of Health and Disease (DOHaD) concept. Food Chem..

