# Supplementary material for: A Review of Dietary Intake of Acrylamide in Humans
Source: Toxics. 2021 Jun 30;9(7):155. doi: 10.3390/toxics9070155 (PMC8309717; doi:10.3390/toxics9070155)
Supplement: Supplementary file 1 [file toxics-09-00155-s001.zip › toxics-1255772-supplementary.pdf]

toxics

Review

A Review of Dietary Intake of Acrylamide in Humans

Clara Amalie Gade Timmermann, Signe Sonne Mølck, Manik Kadawathagedara, Anne Ahrendt Bjerregaard, Margareta Törnqvist, Anne Lise Brantsæter and Marie Pedersen

Table S1. Characteristics of the individual studies that evaluates total dietary intake of acrylamide (AA).

First Author Year	Study Population (*n*)	Dietary Method and Years	AA Food Monitoring Data	Estimated AA Intake µg/kg bw/day	µg/day
**Finland**					
Hirvonen 2010 [1]	Alpha-Tocopherol, Beta-Carotene Cancer Prevention (ATBC) study (RCT of supplementation with alpha-tocopherol/beta-carotene) among male smokers (*n* = 27,111).	Self-administered, modified diet history method with 276 items and frequency of consumption during previous 12 month. Portion size booklet of 122 photographs of foods, five different portion size illustrated (1985–1988).	Obtained from published Finnish data, and chemical analyses conducted particularly for the present study using LC-MS/MS. Data from Sweden and the Netherlands were used for some food items.		P50 (P10–P90)<br>36.8 (21.9–55.7)
Hirvonen 2011 [2]	Diabetes Prediction and Prevention (DIPP) Nutrition Study among children from Tampere 1, 3 and 6 years old (*n* = 1514).	Food consumption data from the DIPP Nutrition Study: Parents and other caregivers recorded the child's food consumption (including type, brand and preparation method), using a 3-day food record that included 1 day of the weekend.	Obtained mainly from published Finnish data.	P50 (P97.5)<br>1y: 0.4 [1]<br>3y: 1.01 (1.95)<br>6y: 0.87 (1.53)	
	FINRISK/FINDIET, a random sample of people from 5 Finnish regions (*n* = 2038).	48-h recall covered all days except Fridays (January-March 2007), 50% of participants also filled in a 3-day food record immediately after the 48h recall and in June–December.		P50 (P97.5)<br>25–44y males: 0.41 (0.87)<br>25–44y females: 0.44 (1.16)	

[1] The estimate for 1-year-olds was not given in text and was thus visually inspected from Figure 3 in the publication.

**Norway**					
Dybing 2003 [3]	Subset of Norwegian children (*n* = 6736).<br><br>Males 9 years (*n* = 1299)<br>Females 9 years (*n* = 1658)<br>Males 13 years (*n* = 1711)<br>Females 13 years (*n* = 2068).<br><br>Subset of Norwegian adult population aged 16-79 years (*n* = 2672).<br><br>Males (*n* = 1291)<br>Females (*n* = 1381).	Obtained from the national food survey NORKOST (2000) based on a 4-day food intake registration in which portions were assigned according to an illustrative book with different portion sizes<br><br>Obtained from the national food survey NORKOST (1997) based on a quantitative frequency questionnaire.	The Norwegian Food Agency (2002) conducted analysis of 30 different food products plus eight brands of coffee bought on the Norwegian market. The food samples were analysed using LC-MS-MS.	P50 (P97.5)<br>Males 9y: 0.23 (1.50)<br>Females 9y: 0.25 (1.10)<br>Males 13y: 0.30 (2.85)<br>Females 13y: 0.28 (2.07)<br><br>P50<br>Males: 0.41 (1.62)<br>Females: 0.42 (1.45)	P50 (P97.5)<br>Males: 29 (125)<br>Females: 24 (88)
Bjellas 2007 [4]	Employees at the Norwegian Institute of Public Health (*n* = 53).<br><br>Non-smokers (*n* = 47)<br>Smokers, actively selected (*n* = 6)	24-h dietary recall with pictures with different portion sizes (2006).	Norwegian Food Safety Authority (2002), the National Food Administration in Sweden (2002), the European Commission (2005), and previous research.		P50 (min–max)<br>Non-smokers: 21 (13–178)<br>Smokers: 26 (12-67)
Bjellas 2007 [5] [2]	As above (*n* = 50).	FFQ covering the last year (2006).	As above		P50 (min-max)<br>Non-smokers: 13.5 (4.1–72.6)<br>Smokers: 18.3 (7.8–32.0)
Brantsæter 2008 [6]	Subsample of pregnant women participating in the Norwegian Mother and Child Cohort study (MoBa): Healthy women referred to Bærum hospital for routine pregnancy ultrasound (*n* = 119).	A semi-quantitative FFQ covering the first four months of pregnancy, and a 4 day food diary (FD) recording weight of all foods and bevarages consumed during three consecutive weekdays and one weekend day (2003-2004).	Obtained from Norwegian Food Safety Authority (2002, 2006), Scientific Committee of the Norwegian Food Control Authority (2002), and the European Union database (2005).	P50 (P99)<br>4-day FD: 0.41 (1.36)<br>FFQ: 0.48 (1.83)	P50<br>4-day FD: 28.5<br>FFQ: 33.7
Duarte-Salles 2013 [7] [3]	Pregnant women from MoBa (as above): Women with singleton births who answered three questionnaires (*n* = 50,651).	FFQ (same as above), 255 items (1999-2008).	Obtained from the Norwegian Food Safety Authority (2002, 2006), Scientific Committee of the Norwegian Food Control	Mean (SD)<br>0.4 (0.2)	Mean (SD)<br>27.1 (13.4)<br><br>Mean (SD), ng/kcal/day<br>11.7 (4.6)

[2] Same population as [4]

[3] Same population as [6]

			Authority (2002), the National Food Administration in Sweden (2002), the European Commission (2005), and previous research	P50 (P75), ng/kcal/day<br>11.1 (14.3)
Kadawathagedara 2018 [8] [4]	Pregnant women from MoBa (as above): Women with singleton births with no malformations (*n* = 51,952).	FFQ (same as above), 225 items (1999-2008).	Obtained from Norwegian, Swedish and European food safety authorities (as above).	P50 (P25–P75)<br>24.7 (18.4–33.2)<br><br>P50 (P25–P75), µg/kcal/day<br>0.011 (0.008–0.014)
**Sweden**				
Svensson 2003 [9]	Swedish National Food Administration Food Survey (*n* = ~1200).	Pre-coded 7-day record book with quantity indications and an illustrated portion guide (1997–1998).	Food samples (>130) were collected from supermarkets in Uppsala, Sweden in 2002. The samples were analysed using LC-MS-MS.	P50 (min–max)<br>27 (0–138)
Mucci 2003 [10]	Population-based case–control study among individuals born in Sweden between 1918 and 1942 and residing in Stockholm for at least 1 month between November 1992 and December 1994 (*n* = 1525).<br><br>Controls (*n* = 538).<br>Cancer of the large bowel (*n* = 591).<br>Cancer of the bladder (*n* = 263).<br>Cancer of the kidney (*n* = 133).	Dietary habits in the 5 years prior to the study were assessed by a semi–quantitative 188 item FFQ (1992–1994).	100+ food samples were analysed at the Swedish National Food Administration (2002).	Mean (SE)<br>Controls: 27.5 (0.6)<br>Large bowel cancer cases 28.6 (0.6)<br>Bladder cancer cases: 29.4 (0.9)<br>Kidney cancer cases: 28.4 (1.2)
Mucci 2004 [11]	Swedish component of an international collaborative population-based case-control study of renal cell cancer. Cases identified through cancer registries and randomly selected age and sex matched controls identified through population registry (*n* = 732).	Face–to–face interviews (1989–1991) based on FFQ asking about usual diet prior to 1987. Information was collected on 11 food items with elevated acrylamide levels.	Obtained from food databases in Sweden and the USA.	P50 (P25–P75)<br>25.9 (20.1–31.9)

[4] Same population as [6]

Mucci 2005 [12]	Women's Lifestyle and Health Cohort (*n* = 43,404).	Semiquantitative FFQ (1991).	The Swedish NFA acrylamide database was used to determine median levels in foods.		Mean (min–max) 25.9 (0–170)
Mucci 2006 [13]	Women from Västmanland County and Uppsala County eligible to participate in a population-based mammography screening program, The Swedish Mammography Cohort (*n* = 61,467).	FFQ covering the previous 6 months, 67 items (1987–1990).	Obtained from the Swedish National Food Administration (2002) and the U.S. Food and Drug Administration (2002).	Mean (P25–P75) 0.38 (0.27–0.47)	Mean (P25–P75) 24.6 (18.7–29.9)
Larsson 2009a [14] [5]	As above (*n* = 61,433)	As above	Obtained from the Swedish National Food Administration and previous research.	Mean (SD) 0.38 (0.17)	P50 (P25–P75) 24.2 (19.9–28.9)
Larsson 2009b [15] [6]	As above (*n* = 61,226)	As above	As above	Mean (SD) 0.38 (0.17)	P50 (P25–P75) 24.2 (19.9–28.9)
Larsson 2009c [16] [7]	As above (*n* = 61,057)	As above	As above		P50 (P25–P75) 24.6 (20.5–29.2)
Wirfält 2008 [17]	A subset of samples from the blood bank of the MDC cohort (Malmö). The samples were selected among non-smokers to obtain a distribution from three exposure categories of AA from food: low, random, and high (*n* = 142).	Information from a 7–day menu book, a 168–item dietary history questionnaire (reference period was the preceding year), and a 1–h interview (focused on details about food preparation and on portion sizes recorded in the menu book) were combined (1991–1996).	Obtained from previous research (Swedish foods).		P50 (min–max) 45 (3.5–189)
Vikström 2012 [18] [8]	Subsample of the above (*n* = 68)	As above	As above	Mean (SD), min–max 0.67 (0.55), 0.04–2.9	(Min–max) (4.5–189)
Larsson 2009d [19]	Men from Västmanland County and Örebro County born between 1918 and 1952 (*n* = 45,306).	FFQ reporting average frequency of consumption of 96 foods and beverages over the past year (1997).	Obtained from the Swedish National Food Administration (1991) and previous research.		P50 (P25–P75) 35.4 (29.6–41.7)

[5] Same population as [13]

[6] Same population as [13]

[7] Same population as [13]

[8] Same population as [17]

Larsson 2009e [20] [9]	As above	As above	As above		Mean (SD), P20–P80<br>36.1 (9.6), 28.3 – 43.4
Wilson 2009a [21]	The Cancer of the Prostate in Sweden (CAPS) study - a population-based case-control study among men (*n* = 2617).<br><br>Controls (*n* = 1118)<br>Cases (*n* = 1499)	Self–administered 261–item FFQ assessing usual intake of foods over the past year (2001–2002).	Obtained from the Swedish National Food Administration (2002).	Range (cases and controls)<br>0.08–1.59<br><br>Mean (SD)<br>Controls: 0.56 (0.20)<br>Cases: 0.54 (0.18)	Range (cases and controls)<br>8–125<br><br>Mean (SD)<br>Controls: 44.5 (14.5)<br>Cases: 43.8 (13.7)
Lin 2011 [22]	Case-control study. Newly diagnosed patients with esophageal cancer and age and sex frequency matched controls selected randomly from the entire Swedish population (*n* = 1438).	Open answer FFQ evaluating the habitual intake 20 years before the interview (in 1994–1997).	Obtained from the Swedish National Food Administration (2002) and previous research.		P50 (P25–P75)<br>34.8 (27.3–44.1)
**Denmark**					
Jakobsen 2016 [23]	The Danish National Survey (children and adults) of Diet and Physical Activity (*n* = 2700).	7 day pre–coded food records (2005–2008).	Obtained from Danish surveys on specific food types and the general monitoring program (2003–2013).	P50 (P99)<br>0.22 (1.42)	
**Republic of Ireland and UK**					
Mills 2008 [24]	Representative sample of adult Irish population (*n* = 958).<br><br>Representative sample of adult UK population (*n* = 1724).	From the North/South Ireland Food Consumption Survey. It was conducted over 7 days and involved the keeping of a food diary detailing each consumption event (1997–1999)<br>From the National Diet and Nutrition Survey for adults. Conducted as in Ireland.	Obtained from the European Union acrylamide monitoring database containing data collected since 2002. Additional data were obtained from the Dublin Public Analyst's Laboratory.	Mean (P97,5)<br>Ireland: 0.59<br>Irish consumers[10]: 0.59 (1.75)<br><br>Mean (P97,5)<br>UK: 0.56<br>UK consumers[11]: 0.61 (1.29)	
**UK**					
Burley 2010 [25]	UK Women's Cohort Study (*n* = 35,372).	A 217–item FFQ (1995–1998)	AA levels from European Commission Institute for	P50 (P25–P75)<br>0.23 (0.15–0.33)	P50 (P25–P75)<br>15 (10–21)

[9] Same population as [19]

[10] Consumer estimate is restricted to only those people in the total population that consume foods containing acrylamide

[11] Consumer estimate is restricted to only those people in the total population that consume foods containing acrylamide

			Reference Materials and Measurements, 2006.		
**The Netherlands**					
Konings 2003 [26]	Non-institutionalised children and adults from the Dutch population (*n* = 6250).	The third Dutch National Food Consumption Survey (NFCS): a 2–day dietary record on 2 consecutive days per person. The survey was distributed equally over the 7 days of theweek and over 1 year (except holidays). The amount eaten was weighed accurately (1998).	Collected 151 food samples that were analysed using LC–MS–MS. AA in coffee was based on data from Swiss authorities.	P50 (P99.99)<br>Population: 0.2 (2.7)<br>1–6y: 0.3 (3.8)<br>7–18y: 0.2 (3.2)	
Boon 2005 [27] [12]	As above, children and adults (*n* = 6250)<br>1-6 year-old children (*n* = 530).	As above	From the Institute for Reference Materials and Measurements (IRMM) of the European Commission's Directorate General Joint Research Centre (JRC) database of AA levels from different countries (2003–2004).	P50 (P95)<br>Population: 0.5 (1.2)<br>1–6y: 1.1 (2)	
Hogervorst 2008a [28]	The Netherlands Cohort Study. on diet and cancer, adults (NLCS, *n* = 4438).	FFQ with 150 food items covering habitual intake, in terms of frequency and, for most foods, portion size, of foods consumed by the participant during the year preceding baseline.	AA levels from Dutch Food and Consumer Product Safety Authority (2002). In 2005, another series of foods was analyzed to specifically accommodate the estimation of acrylamide intake of the NLCS cohort	Mean (SD)<br>Subcohort: 0.30 (0.18)	Mean (SD)<br>Subcohort: 21.8 (12.0) [13]
Hogervorst 2008b [29] [14]	As above (*n* = 4438)	As above	As above	Mean (SD)<br>Subcohort: 0.30 (0.18)	Mean (SD)<br>Subcohort: 21.8 (12.1) [15]

[12] Same population as [26]

[13] The study also reports AA levels for different types of cancer patients

[14] Same population as [28]

[15] The study also reports AA levels for different types of cancer patients

Hogervorst 2009a [30] [16]	As above (*n* = 4438)	As above	As above	Mean (SD)<br>Subcohort: 0.30 (0.18)	Mean (SD)<br>Subcohort: 21.8 (12.1) [17]
Hogervorst 2009b [31] [18]	As above (*n* = 4438)	As above	As above	Mean (SD)<br>Subcohort:<br>Males: 0.29 (0.16)<br>Females: 0.32 (0.19)	Mean (SD)<br>Subcohort:<br>Males: 22.6 (12.2)<br>Females: 21.0 (11.9) [19]
Hogervorst 2014 [32] [20]	As above (*n* = 3988)	As above	As above	Mean (SD)<br>Subcohort:<br>Males: 0.29<br>Females: 0.32	Mean (SD)<br>Subcohort:<br>Males: 22<br>Females: 21 [21]
Bongers 2012 [33] [22]	As above (*n* = 4438)	As above	As above	Mean (SD)<br>Subcohort:<br>Males: 0.29 (0.16)<br>Females: 0.32 (0.19)	Mean (SD)<br>Subcohort:<br>Males: 23 (12)<br>Females: 21 (12) [23]
Lipunova 2017 [34] [24]	As above (*n* = 4438)	As above	As above		Mean (SD)<br>Subcohort:<br>Males: 22.6 (12.2)<br>Females: 21.0 (11.9) [25]
Schouten 2009 [35] [26]	Case-control study within the NLCS, adults (*n* = 2456, cases = 434, subcohort = 2022).	As above	As above		Mean (SD)<br>Subcohort: 21.8 (12.1) [27]

[16] Same population as [28]

[17] The study also reports AA levels for different types of cancer patients

[18] Same population as [28]

[19] The study also reports AA levels for different types of cancer patients

[20] Same population as [28]

[21] The study also reports AA levels for different types of cancer patients

[22] Same population as [28]

[23] The study also reports AA levels for different types of cancer patients

[24] Same population as [28]

[25] The study also reports AA levels for different types of cancer patients

[26] Same population as [28]

[27] The study also reports AA levels for different types of cancer patients

Hogervorst 2007 [36] [28]	Women from the NLCS (*n* = 2589).	As above	As above	Mean (SD)<br>0.32 (0.19)	Mean (SD)<br>21.0 (11.9)
Pedersen 2010 [37] [29]	As above (*n* = 2247).	As above	As above	P50 (P10–P90)<br>0.26 (0.14–0.57)	P50 (P10–P90)<br>17.9 (9.5–36.8)
Hogervorst 2016 [38] [30]	Case-cohort study among women in the NLCS (*n* = 1838, cases = 364, subcohort = 1474).	As above	As above		Mean (SD)<br>Cases: 21.3 (12.7)<br>Subcohort: 20.9 (11.7)
Hogervorst 2017 [39] [31]	As above	As above	As above		Mean (SD)<br>Cases: 21.9 (13.1)<br>Subcohort: 20.9 (11.8)
Hogervorst 2019 [40] [32]	As above	As above	As above		Mean (SD)<br>Cases: 20.6 (11.3)<br>Subcohort: 21.0 (11.8)
Perloy 2018 [41] [33]	Case-cohort study amnong men in the Netherlands Cohort Study (NLCS) (*n* = 2556 , cases = 948, subcohort = 1608).	As above	As above		Mean (SD)<br>Cases: 22.9 (12.2)<br>Subcohort: 22.4 (11.9)
Konings 2010 [42]	Segment of the Dutch adult population (*n* = 122).	From The National Institute for Public Health and the Environment (RIVM): Participants were asked to provide information about the quantity of the various foods and drinks consumed during 24h sampling period (2004).	Obtained from the Food and Consumer Product Safety Authority (VWA) that had analyzed foods on the Dutch market in 2002 and 2005.	Mean (SD)<br>0.45 (0.49)	
**Belgium**					
Matthys 2005 [43]	Adolecents (13–18 years) from the region of Ghent (*n* = 341).	A 7–day estimated food record method (semi–structured diary) was used to quantify food and nutrient intake based on 745 different food items (1997).	In 2003, the Belgian Federal Agency for the Safety of the Food Chain collected 150 food items from different supermarkets and	P50 (P1–P99)<br>0.51 (0.11–1.41)	

[28] Same population as [28]

[29] Same population as [28]

[30] Same population as [28]

[31] Same population as [28]

[32] Same population as [28]

[33] Same population as [28]

			restaurants. The acrylamide analysis was conducted using LC–MS–MS		
Claeys 2010 [44]	Belgian Food Consumption Survey (BFCS) among persons >15 years of age (*n* = 3215).	2 repeated non–consecutive 24h recall interviews and a self–administered FFQ.	Based on AA monitoring data of the Belgian Federal Agency for the Safety of the Food Chain (FASFC).	P50 (P25 – P97.5, P99)<br>0.2 (0.10 – 1.58, 2.26)	
**Germany**					
Hilbig 2004 [45]	Infants, children and adolescents from the DONALD (Dortmund Nutritional and Anthro pometric Longitudinally Designed) study.<br><br><1y (*n* = 365)<br>1–6y (*n* = 1121)<br><br>7–18y (*n* = 1351)	DONALD study: 3–day–weighed dietary records.	Obtained from the German Federal Office for the Protection of the Consumer and Food Safety, which compiled more than 1500 data on acrylamide analysis of various foods until the end of March 2002.	P50 (P10–P90) [34]<br>DONALD <1 y: 0.19 (0.01–0.39)<br>DONALD 1–<7 y: 0.31 (0.08–0.90)<br>DONALD 7–<19y: 0.20 (0.03–0.67)	
	Children from several duplicate studies carried out at the Department of Hygiene–, Social and Environmental Medicine, Ruhr–University Bochum (RUB), 1–7y (*n* = 119)	RUB studies: 3– or 7–day estimated dietary records. Estimated food quantities were recalculated by use of standard tables from the DONALD study.		RUB (1–<7y): 0.46 (0.16–1.19)	
**Poland**					
Mojska 2010 [46]	Polish population, adults and children (*n* = 4134).<br>1–6 years (*n* = 284)<br>7–18 years (*n* = 957)<br>Adults (*n* = 2893).	Obtained from the 'Household Food Consumption and Anthropometric Survey in Poland': a 24–h dietary recall carried out using an 'Album of Photographs of Food Products and Dishes' (2000).	The tests covered food samples taken in the 2005–2007 period by the sanitary inspection employees at randomly selected stores, catering establishments such as bars and restaurants all over Poland. Altogether 225 samples of foodstuffs were tested using GC–MS/MS.	P50 (P5–97.5)<br>Population: 0.27 (0.07–2.12)<br>1–6y: 0.35 (0.06–4.54)<br>7–18y: 0.33 (0.07–3.58)<br>Adults: 0.26 (0.07–1.13)	Mean (SD)<br>Population: 23.3 (30.5)<br>1–6y: 13.24 (24.4)<br>7–18y: 26.7 (43.0)<br>Adults: 23.1 (25.8)
Zając 2013 [47]	Random individuals (adults and children) from random	Semi–quantitative FFQ conducted face to face by		Mean (SD), P95<br>Population: 0.85 (0.82), 1.70	

[34] AA levels were calculated for different scenarios, we report results calculated using median content of AA in foods.

	households from 3 southern Polish provinces chosen for survey with respect to different age groups (*n* = 1470)<br><br>6–12 years (*n* = 300).<br>13–19 years (*n* = 296)<br>20–30 years (*n* = 296)<br>31–41 years (*n* = 278)<br>42–60 years (*n* = 300)	trained interviewers, 164 different food items. Photographic Album of Products and Dishes (2011–2012).	European acrylamide monitoring database from June 2006.	P50 (P25–P95)<br>6–12y: 1.36 (0.83–2.86)<br>13–19y: 0.62 (0.35–3.00)<br>20–30y: 0.56 (0.41–1.15)<br>31–41y: 0.52 (0.40–1.10)<br>42–60y: 0.35 (0.33–1.12)	
Wyka 2015 [48]	Teenagers (16–18 years) from urban environment – randomly selected pupils from four schools (*n* = 261).<br><br>Males, 43% (*n* = ~112)<br>Females, 57% (*n* = ~149)	A 7–day food record diary (consecutive days excluding weekend and holidays). Food records (n = 1827) were obtained face–to–face by trained interviewers.<br>The size of food rations was estimated with the use of an 'Album of Photographs of Food Products and Dishes' (2012) (2010–2011).	Obtained from the national database of the Institute of Food and Nutrition in Warsaw.	P50 (P95)<br>Males: 0.13 (1.18)<br>Females: 0.09 (1.04)	
Mojska 2016 [49]	Healthy women who gave birth at term to healthy babies in one obstetric clinic in Warsaw (*n* = 93)<br>Ten of the women recieved drip infusion only.	24–h Dietary Recall while at the hospital on day 2–5 after birth. Face–to–face interview. The portion size was verified with the use of an 'Album of Photographs of Food Products and Dishes' (2012)	Same as Mojska 2010 [46]	P50 (min–max)<br>0.10 (0.00–0.66)	P50 (min–max)<br>7.0 (0.0–51.3)
**France**					
Sirot 2012 [50]	National data on children 3–17 year old (*n* = 1444)<br><br><br><br><br><br><br><br>National data on adults (*n* = 1918)	Consumption data from the second national consumption survey (INCA2). Subjects completed a 7–day food record diary (consecutive days). Portion sizes were estimate through photographs (2005–2007)	French total diet study. Foods were sampled in several French regions two times (winter–summer or autumn spring). A total of 2280 products were purchased and grouped into 192 analytical samples analyzed using LC–MS	Mean (SD), P95<br>3–17y: 0.69 (0.58), 1.80<br><br>Mean, P95<br>3–6y: 0.89, 1.86<br>7–10y: 0.80, 2.00<br>11–14y: 0.57, 1.51<br>15–17y: 0.45, 1.17<br><br>Mean (SD), P95<br>Adults: 0.43 (0.33), 1.02	

Mancini 2015 [51] [35]	As above (*n* = 1918)	As above	Second French Total Diet Study (TDS2) carried out from 2007 to 2009	P50 (P99)<br>0.35 (1.42) [36]	
Chan–Hon–Tong 2013 [52]	Pregnant women from the EDEN cohort.<br>The year before pregnancy (*n* = 1861).	FFQ covering diet in the year before pregnancy and during the last three months of pregnancy: 137 food items, sizes were determined using pictures or assumed to be a standard portion for the French adult population.	Second French Total Diet Study (TDS2) carried out from 2006 to 2010. Food products representative of the French diet were bought in eight regions and grouped into 1319 food samples corresponding to 212 core foods collected during different seasons.	Mean (P95) [37]<br>Before pregnancy: 0.40 (0.97)<br>Third trimester: 0.29 (0.71)	
Kadawathagedara 2016 [53] [38]	As above (*n* = 1471).	FFQ covering the last three months of pregnancy (see above).	As above	P50 (min–max)<br>0.26 (<0.01–2.59)	P50 (min–max)<br>19.2 (0.16–183.5)
**Italy and Switzerland**					
Pelucchi 2006 [54]	Adult controls from an integrated series of hospital–based case–control studies from northern Italy + Vaud in Swizerland with the same design, questionnaire and inclusion criteria. Cases were cancer patients and controls were patients with acute non–neoplastic conditions.<br><br>Study 1 controls (*n* = 1772)<br>Study 2 controls (*n* = 1066)<br>Study 3 controls (*n* = 4765)<br>Study 4 controls (*n* = 1297)	FFQ with 78 items assessing the two years before diagnosis (1991–2002).	Obtained from WHO and the Swiss Federal Office of Public Health		P50 (P20–P80)<br>Study 1 controls: 24.0 (12.9–40.4)<br><br>Study 2 controls: 23.8 (13.2–39.6)<br>Study 3 controls: 23.4 (12.4–39.6)<br>Study 4 controls: 23.8 (13.1–38.2)

[35] Same population as [50]

[36] Estimated exposure was calculated using 3 different models, we report results from the model based on observed individual mean only

[37] Estimated exposure was calculated using different approaches for treating values below LOD/LOQ, we report results where values below LOD were replaced by the LOD and the values below LOQ were replaced by the LOQ

[38] Same population as [52]

	Study 5 controls, women only (*n* = 3122)<br>Study 6 controls, Italian women only (*n* = 2411)<br>Study 7 controls, Italian men only (*n* = 1451).				Study 5 controls: 20.6 (10.6–34.3)<br>Study 6 controls: 20.3 (10.1–32.4)<br>Study 7 controls: 22.9 (12.4–36.4)
**Italy**					
Pelucchi 2007 [55] [39]	Renal cell cancer patients and controls patients with acute non–neoplastic conditions from part of the same study area as above (*n* = 2301).	As above	As above	Mean<br>0.48	P50 (P25–P75)<br>31.2 (20.4–44.1)
Pelucchi 2011 [56] [40]	Pancreatic cancer patients and control patients from province of Pordenone, which is part of the same study area as above (*n* = 978).<br>Controls (*n* = 652)<br>Cases (*n* = 326).	FFQ with 78 items assessing the two years before diagnosis (1991–2008).	AA levels from World Health Organization (WHO) and the Agence Francaise de Sécurite´ Sanitaire des Aliments.		Mean<br>Controls: 32.19<br>Cases: 33.51
Pelucchi 2016 [57] [41]	Hospital controls from a endometrial cancer case–control study among women from three Italian provinces in the same study area as above (*n* = 908)	FFQ with 78 items assessing the two years before .diagnosis/hospital admission (1992–2006)	As above		Mean (SD), P20–P80<br>29.8 (16.1), 17.7–39.2
**Cyprus**					
Kafouris 2018 [58]	Child Health Database for adolescents 11–15 years (*n* = 303).	Child Health Database for adolescents in Cyprus, which is part of the EFSA Comprehensive Food Consumption Database. A 3–day food record (2003).	Samples of potato crisps, French fries, bread, breakfast cereals, biscuits, crackers, crispbread, roasted and instant coffee were collected from retail market by the Public Health Services of the Ministry of Health of Cyprus and	Mean, P95<br>0.8, 1.8	

[39] Same population as [54]

[40] Same population as [54]

[41] Same population as [54]

			analyzed using UPLC–MS/MS.		
**Europe**					
Obón–Santacana 2013 [59]	The European Prospective Investigation into Cancer and Nutrition (EPIC) is a multicenter prospective cohort study that includes participants from 23 research centers in 10 European countries (*n* = 477,308).<br>Denmark, males (*n* = 26,294)<br>Denmark, females (*n* = 28,722)<br>France, females (*n* = 67,382)<br>Germany, males (*n* = 21,172)<br>Germany, females (*n* = 27,411)<br>Greece, males (*n* = 10,807)<br>Greece, females (*n* = 15,225)<br>Italy, males (*n* = 14,029)<br>Italy, females (*n* = 30,512)<br>Norway, females (*n* = 35,169)<br>Spain, males (*n* = 15,148)<br>Spain, females (*n* = 24,854)<br>Sweden, males (*n* = 22,308)<br>Sweden, females (*n* = 26,375)<br>The Netherlands, males (*n* = 9639)<br>The Netherlands, females (*n* = 26,866)<br>UK, males (*n* = 22,852)<br>UK, females (*n* = 52,543)	Country–specific validated food intake questionnaires (1992–1998)	Obtained from the European Community Institute for Reference Materials and Measurements database. Methods were based on either liquid or gas chromatography coupled to mass spectrometry. The IRMM database includes AA levels in foods mainly from Austria, Germany, Greece, Ireland, The Netherlands, the UK, and from the food industry.		P50, mean (SD)<br>All: 23.3, 26.2 (14.8)<br>Denmark, males: 42.3, 43.2 (13.8)<br>Denmark, females: 34.5, 35.5 (11.7)<br>France, females: 19.1, 20.4 (8.8)<br>Germany, males: 28.0, 30.5 (13.5)<br>Germany, females: 22.4, 24.5 /11.2)<br>Greece, males: 21.6, 24.0 (13.2)<br>Greece, females: 17.5, 19.0 (9.0)<br>Italy, males: 10.0, (11.5 (6.7)<br>Italy, females: 9.6, 10.9 (6.1)<br>Norway, females: 17.4, 17.9 (6.5)<br>Spain, males: 24.9, 27.8 (16.0)<br>Spain, females: 18.3, 20.5 (12.1)<br>Sweden, males: 27.0, 29.2 (12.8)<br>Sweden, females: 20.6, 22.4 (9.7)<br>The Netherlands, males: 36.1, 38.4 (16.3)<br>The Netherlands, females: 29.1, 31.0 (13.6)<br>UK males: 36.5, 39.1 (17.8)<br>UK females: 30.6, 32.9 (15.22)
Freisling 2013 [60] [42]	Age- and gender-stratified random sample of EPIC (*n* = 36,994, 8% of entire EPIC).	24h-dietary recall in telephone (Norway) or face-to-face (all other countries) interviews (1995-2000). Portions size was	As above		Mean, study sites with highest and lowest values:<br>Males:<br>15, Navarra (Spain)

[42] Same population as [59]

		assessed using picture book.<br>Energy intake was asessed.			48, Aarhus (Denmark)<br>Females:<br>12, Ragusa (Italy)<br>41, Aarhus (Denmark)
Ferrari 2013 [61] [43]	Assessing validity of DQs and DRs in a subgroup of EPIC (*n* = 510).	Habitual dietary intakes was assessed at<br>baseline with different dietary questionnaires (France, the Netherlands, Germany, Greece, and Italy, except Naples), a diet history questionnaire (Spain), a modified diet history methodology combining a quantified dietary frequency questionnaire, a 7–day menu book and a 1–h interview (Sweden), or a semi–quantitative food frequency questionnaire (United Kingdom and Naples).<br>24–h dietary recall was collected from a subsample (8 %) of each cohort.	As above		Geometric mean (P10–P90)<br><br>DQ: 24.7 (11.6–50.4)<br>DR: 21.8 (7.1–59.5)
Obón-Santacana 2014 [62] [44]	Women in EPIC (*n* = 301,113).	Country-specific validated food intake<br>questionnaires refering to the previous 12 months (1992–1998).	As above	Mean (SD)<br>All: 0.4 (0.2)<br>France: 0.4 (0.2)<br>Italy: 0.2 (0.1)<br>Spain 0.3 (0.2)<br>U.K: 0.5 (0.3)<br>Netherlands 0.5 (0.2)<br>Greece: 0.3 (0.1)<br>Germany 0.4 (0.2)<br>Sweden: 0.3 (0.2)<br>Denmark: 0.5 (0.2)<br>Norway: 0.3 (0.1)	Mean (SD)<br>23.7 (12.0)<br>18.3 (6.6)<br>8.8 (5.7)<br>21.3 (10.3)<br>33.4 (13.1)<br>31.7 (12.1)<br>19.8 (7.2)<br>25.3 (9.7)<br>23.6 (8.2)<br>35.5 (10.2)<br>20.6 (5.8)

[43] Same population as [59]

[44] Same population as [59]

Lujan-Barosso 2014 [63] [45]	EPIC (*n* = 477,308).	As above	As above		P50 (min–max)<br>23.3 (0–261.4)
Obón-Santacana 2015 [64] [46]	Women in EPIC (*n* = 325,006).	As above	As above	P50 (P25–75)<br>0.3 (0.2–0.5)<br>France: 0.3 (0.2–0.4)<br>Italy: 0.2 (0.1–0.2)<br>Spain: 0.3 (0.2–0.4)<br>UK: 0.5 (0.3–0.7)<br>Netherlands: 0.4 (0.3–0.6)<br>Greece: 0.3 (0.2–0.3)<br>Germany: 0.3 (0.2–0.4)<br>Sweden: 0.3 (0.2–0.4)<br>Denmark: 0.5 (0.4–0.6)<br>Norway: 0.3 (0.2–0.3)	P50 (P25–75)<br>21.9 (16.0–29.8)<br>17.7 (14.0–21.9)<br>8.6 (5.4–11.8)<br>19.5 (14.1–26.2)<br>31.2 (24.2–39.7)<br>29.7 (23.1–38.0)<br>18.9 (15.3–23.1)<br>23.6 (19.1–29.7)<br>22.6 (18.7–27.0)<br>34.7 (28.4–41.5)<br>17.4 (13.6–21.5)
Obón-Santacana 2017 [65] [47]	EPIC except Norway and Denmark, post menstrual nonsmoking women (*n* = 801).	Country specific dietary questionnaires (1992–1998).	As above		P50 (P25–75)<br>20.3 (13.5–29.9)
**Turkey**					
Cengiz 2013 [66]	Survey covering toddlers 1-3 years from the Uncalı province of Antalya (*n* = 302).	24-h diet recall (types and amounts) by trained interviewer (2012).	Detailed information about brands and types of consumed foods was provided in the questionnaire report, and the baby food samples were collected according to the brands and types described in the report. Analyzed by GC-MS.	P95 (min–max)<br>3.76 (0.06-6.41)<br><br>Mean (SD)<br>All: 1.43(1.05)<br>1y: 1.68 (1.10)<br>1.5y: 1.61 (0.91)<br>2y: 1.19 (1.90)<br>2.5y: 1.28 (1.14)<br>3y: 1.16 (0.78)	
**Iran**					
Nematollahi 2020 [67]	The Tehran Lipid and Glucose Study (TLGS). A society–based forthcoming (cohort) study, accomplished on a sample of populations in 13 regions of Tehran (*n* = 7,291).	FFQ containing approximately 150 food items (2017–2018).	Food products collected from the Tehran market was investigated by the aid of a dispersive liquid liquid microextraction (DLLME) system coupled with gas	P50 (P5–P95)<br>3–10y: 1.53 (0.65–3.90)	Mean (SD)<br>43.9 (24.5)

[45] Same population as [59]

[46] Same population as [59]

[47] Same population as [59]

	3–10 years (n = 228)<br>11–17 years (n = 412)<br>18–60 years (n = 5439)<br>61–96 years (n = 1212).		chromatography–mass spectrometry (GC–MS).	11–17y: 0.86 (0.35–2.24)<br>18–60y: 0.51 (0.21–1.35)<br>61–96y: 0.43 (0.16–1.22)	55.1 (29.5)<br>44.8 (26.9)<br>37.1 (25.0)
**China**					
Zhou 2013 [68]	Survey in 12 provinces/municipalities	3-day household dietary survey and 24-h recall collected by Chinese Center for Disease Control and Prevention (Chinese CDC)	Food samples were purchased from local markets, grocery stores, and local farms and analysed using LC-MS/MS	Mean (P95)<br>0.29 (0.49)	
Gao 2016 [69]	(5th TDS) General population, 20 provinces, 2/3 of the population.	3-d household dietary survey and 24-h diet recalls (2009–2012).	The levels of acrylamide in 240 food composite samples from the 5th Chinese Total Diet Study (TDS) were measured using an LC-MS/MS method.	P50<br>0.32	
Chen 2008 [70]	Chinese population	Dietary consumption survey data for the Chinese population (2004).	Foods bought at local supermarkets and stores in Beijing in 2005 and 2006. The 349 samples covered 16 categories of popular foods in China. Analyzed using LC-MS/MS	P50 (P97.5)<br>0.20 (1.50)	Mean<br>22.9
**Hong Kong**					
Wong 2014 [71]	Hong Kong population-based Food Consumption Survey among adults (n = 5008).	Two non-consecutive 24-h dietary records (2005–2007).	A total of 150 commonly consumed food items were selected for the study, based on the food consumption pattern of the Hong Kong population obtained from the FCS. Analyzed using LC-MS/MS.	Mean (P95)<br>0.213 (0.538)	
Liu 2017a [72]	Mr. and Ms. OS Hong Kong study - volunteering elderlies (n = 4000).	FFQ containing 329 food items (2001-2003).	Obtained from the database of the 1st Hong Kong Total Diet Study (HKTDS)	Mean of Q1–Q4<br>0.14–0.41	Mean (SD)<br>14.6 (8.2)

Liu 2017b [73] [48]	As above (*n* = 2534, males = 724, females = 1810)).	As above	As above		Mean (SD)<br>Males: 16.1 (7.5)<br>Females: 13.3 (7.8)
**Korea**					
Lee 2020 [74]	Korean National Health and Nutrition Examination Survey (KNHANES) among children and adults.	Korean National Health and Nutrition Examination Survey (KNHANES) conducted over 3 years (2007–2009).	485 samples of food products were collected from randomly selected local supermarkets, stores, and fast-food restaurants all over Korea in 2011. Analyzed using LC-MS/MS.	P50 (P95), max<br>Population: 0.08 (0.12), 0.15<br>< = 2y : 0.12 (0.37), 0.66<br>3-6y: 0.11 (0.29), 0.49<br>7-12y: 0.06 (0.13), 0.19<br>13-19y: 0.06 (0.12), 0.19<br>20-64y: 0.08 (0.14), 0.16<br>> = 65y: 0.06 (0.10), 0.11	
Jeong 2020 [75]	KNHANES among children and adults (*n* = 32,034).<br>3–5 years (1346)<br>6–11 years (2600)<br>12–18 years (2412)<br>19–64 years (18,715)<br>65–80 years (6961).	KNHANES - 24h dietray recall (2013–2017).	Food products, mostly from local supermarkets, were purchased and analyzed using HPLC-MS/MS.	P50 (P95)<br>Population: 0.04 (0.30)<br>3-5y: 0.07 (0.84)<br>6-11y: 0.05 (0.64)<br>12-18y: 0.03 (0.46)<br>19-64y: 0.04 (0.25)<br>65-80y: 0.02 (0.11)	
**Japan**					
Nagata 2015 [76]	Non-pregnant women participating in medical health check-up program provided by a general hospital in Gifu, Japan (*n* = 393).	FFQ (169 items) over the course of one year (2003–2006).	Obtained from the Japanese ministries of Agriculture, Forestry and Fisheries and of Health, Labour and Welfare and all other available data from published articles on acrylamide concentrations in Japanese foods.		Mean (SD), min–max<br>24.1 (11.1), 7.5–127.3
Nagata 2018 [77]	Children 3–6 years e recruited from 2 preschools in Aichi, Japan (*n* = 428).	3-day diet record, food weighted (2006).	AA levels from The Japanese ministries of Agriculture, Forestry, and Fisheries and of Health, Labor, and Welfare.	Mean (SD), min–max<br>1.00 (0.55), 0.13–4.71	
Kawahara 2018 [78]	National Health and Nutrition Survey (NHNS)	NHNS data: 1-day household dietary records (2012).	As above	P50 (95th percentile)[49]<br>Population: 0.147 (0.226)	

[48] Same population as [72]

[49] This paper used two different calculation methods. We report results from the method in which concentration of acrylamide in each food groups were assumed to range between zero and observed maximum value.

	among children and adults (*n* = 24,293).			Mean<br>1–6y: 0.41<br>7–14y: 0.29<br>15–29y: 0.16<br>30–44y: 0.16<br>45–59y: 0.15<br>60+ y: 0.12	
Yamamoto 2018 [79]	Cross-sectional dietary assessment among adults (*n* = 14).	Diet record (DR), collected two conservative days and FFQ covering the previous year (2014–2015).	AA levels from national database.	P50 (min–max)<br>DR: 0.18 (0.06–0.71)<br>FFQ: 0.11 (0.05–0.26)	
Kotemori 2018a [80]	Japan Public Health Center-based (JPHC) prospective study among adults 45–74 years.<br><br>Cohort 1 (*n* = 215)<br><br>Cohort 2 (*n* = 350)	28-day (14-day for participants in Okinawa) dietary records and 1 or 2 FFQs (1994–1996).	AA levels from previous Japanese studies combined with the Standard Tables of Food Composition in Japan, Fifth Revised and Enlarged Edition (5th FCT).	P50 (5th–95 percentile)<br>Cohort 1<br>DR: 0.10 (0.05–0.23)<br>FFQ: 0.10 (0.03–0.25)<br><br>Cohort 2<br>DR: 0.11 (0.06–0.23)<br>FFQ: 0.12 (0.04–0.25)	P50 (5th–95 percentile)<br>Cohort 1<br>DR: 5.9 (3.0–13.4)<br>FFQ: 6.1 (2.2–13–7)<br><br>Cohort 2<br>DR: 6.6 (3.3–14.1)<br>FFQ: 6.5 (2.4–13.6)
Kotemori 2018b [81] [50]	Women from the JPHC (*n* = 47,185).	FFQ with 138 items (1995).	Obtained from AA content database developed from published reports of measurements of common Japanese foods.	Mean (SD)<br>0.14 (0.13)	P50 (P25–P75)<br>6.3 (4.5–8.8)<br><br>Mean (SD)<br>7.1 (3.7)
Kotemori 2018c [82] [51]	As above (*n* = 48,910)	As above	As above	Mean (SD)<br>0.14 (0.13)	Mean (SD), min-max<br>7.0 (3.7), 0.0-5.1
Liu 2019 [83] [52]	JPHC (*n* = 87,628)	FFQ during the previous year (1995–1998).	Values of acrylamide content in common Japanese foods from previous research.	Mean (SD)<br>0.13 (0.16)	Mean (SD)<br>6.8 (3.8)
Nagata 2019 [84]	Pregnant women (*n* = 204)	5–day diet record approximately the 29th week of pregnancy. All	AA levels from Japanese reports.	Mean (SD), min–max<br>0.34 (0.17), 0.10–1.08	Mean (SD)<br>19.6 (9.9)

[50] Same population as [80]

[51] Same population as [80]

[52] Same population as [80]

		food consumed were weighted (2000–2001).			
**Australia**					
Croft 2004 [85]	Australian population ≥ 2 years (n = 12,655).	Australian food consumption data derived from the 1995 National Nutrition Survey (NNS) - used a 24-h food recall methodology.	LC/MS/MS of composite samples from a survey of more than 100 carbohydrate-based foods in the Australian diet sampled in the Sidney area.	Mean (P95)<br>Population: 0.4 (1.4)	Mean (P95)<br>22 (73)
	2-6-year-old children (n = 954)			2-6y: 1.0 (3.2)	19 (59)
**Canada**					
Normandin 2013 [86]	Montreal Island, Nonsmoking adolescents 10-17 years (n = 196).	2-day food diary and FFQ covering the last month (2009–2010).	AA levels was measured using LC-MS/MS in foods reported to be most often consumed by the participants. (on the basis of answers to the dietary questionnaires).	P50 (min–max)<br>DR: 0.29 (0.00–5.78)<br>FFQ: 0.17 (0.02–0.80)	
Brisson 2014 [87] [53]	As above (n = 195).	As above<br>2-day food diary (N = 195)<br>FFQ (N = 185).	As above	P50 (min–max)<br>DR: 0.29 (0.00–5.78)<br>FFQ: 0.17 (0.02–0.80)	
**USA**					
Wilson 2009b [88]	Women from Nurses' health study II (n = 85,092, subgroup = 296).	FFQ for food consumption previous 12 months. For each food item a portion size was given (1989–2005).	When possible, obtained from the U.S. Food and Drug Administration (FDA)'s Exploratory Analysis of Acrylamide in Foods, which began in 2002 and continued as part of the FDA's Total Diet Study.		Mean (SD)<br>Full cohort: 20.1 (8.4)<br>Subgrop: 19.3 (7.9)
Wilson 2009c [89]	As above (n = 90,628)	As above	As above	Mean of Q1–Q5<br>0.17–0.58	Mean of Q1–Q5<br>10.8–37.8
Wilson 2010 [90]	As above (n = 88,672)	As above (1976–2006)	As above	Mean of Q1–Q5<br>0.13–0.42	Mean of Q1–Q5<br>9–26
Hogervorst 2013 [91]	As above (n = 1987, premenopausal = 1300, postmenopausal = 687).	As above	As above		Mean of Q1–Q4<br>Premenopausal: 11.6–35<br>Postmenopausal: 8.0–26.8

[53] Same population as [86]

Graff 2018 [92] [54]	As above (n = 88,767) +<br><br>The Health Professionals Follow-up Study (HPFS), a prospective cohort study among male health professionals (n = 47,797)	Semi-quantitative FFQ (1980–2014)<br>Semi-quantitative FFQ (1986–2014)	As above		P50 (P25–P75)<br>15.1 (10.2–20.8)<br>20.2 (14.6–27.5)
Wilson 2012 [93]	HPFS as above (n = 47,896)	FFQ with >130 items covering the past 12 months (1986–2002).	As above		P50 (P10–P90)<br>21 (12–35)
Doerge 2008 [94]	Estimation of the probable AA intake in the USA population, children and adults.	Food and beverage consumption values were taken from the U.S. Department of Agriculture (USDA) Continuing Survey of Food Intake by Individuals (CSFII, 1994–1996 and 1998 Supplemental Children's Survey).	Obtained from FDA (20022004) as well as surveys of selected foods from FDA's Total Diet Study (20032006).	Mean (P90)<br>Population: 0.44 (0.95)<br>25y: 1.1 (2.3)	
Tran 2010 [95]	National Health and Nutrition Examination Survey (NHANES) among children and adults 2003–2004 (n = 5306).<br><br>Children 3–12 years (n = 1019)<br>Teenagers 13–19 years, males (n = 561)<br>Teenagers 13–19 years, females (n = 640)<br>Adults, males (n = 1408)<br>Adults, females (n = 1678)	FFQ (152 categories of food) covering the past 12 months. Information about individual portion size was derived from a 24-h dietary recall interview (2003–2004).	AA levels from FDA	P50 (P25–P95)<br>Population: 0.33 (0.20–1.15)<br>3–12y: 0.69 (0.41–2.39)<br>Males, 13–19y: 0.46 (0.27-1.44)<br>Females, 13–19y: 0.34 (0.18–1.23)<br>Males: 0.33 (0.21–0.91)<br>Females: 0.27 (0.16–0.75)	P50 (P25–P97)<br>23.3 (14.6–63.1)<br>21.5 (14.2–53.8)<br>32.3 (19.3–92.2)<br>21.6 (11.0–72.4)<br>28.3 (18.1–71.4)<br>19.5 (40.8–50.1)
Katz 2012 [96] [55]	Western and guideline based diets were modeled from day one of teenage (13–19 years) diets reported in NHANES 2003–2004 (n = 1899).	As above	As above	Mean (SD), P99.9<br>0.44 (0.003), 0.86	

[54] Part of the population is the same as [91] and part of the population is the same as [93]

[55] Same population as [95]

Duke 2018 [97] [56]	NHANES 2003–2004, 3+ years (*n* = 9033).	24–hour dietary interview, self–reported if above 12 years (2003–2004).	As above	Geometric mean<br>Children: 0.38<br>Adolescent: 0.18<br>20-59y: 0.15<br>60+y: 0.14	
Abt 2019 [98] [57]	Data from NHANES 1999–2014 and from NPD group, Inc. National Eating Trends – Nutrient Intake Database.<br><br>Individuals >2 years<br><br><br>Individuals <2 years	2 day food survey (1999–2014). For those older than 2 years 14 day food consumption data was also used (2011–2012).	In 2011 and 2015, 1309 and 1208 food products, respectively, were purchased at retail markets or restaurants throughout the USA and analyzed using LC-MS/MS..	Mean (P90)<br>0.36 (0.86) based on 2-day consumption data<br>0.26 (0.53) based on 14- day consumption data<br><br><br><br>1.42 (3.02)	
McCullough 2019 [99]	The prospective Cancer Prevention Study (CPS)-II Nutrition Cohort (*n* = 102,154).	FFQ with 152 items covering the past year (1999–2013).			Mean for Q1–Q4<br>13.4–33.0<br><br>Mean (SD)<br>Males: 25.7 (10.4)<br>Females: 19.4(6.9)
**Brazil**					
Arisseto 2009 [100]	Adolescents 11–17 years old.	24 h recall dietary asked by a trained interviewer.	Different brands and batches of selected foods were purchased at supermarkets, fast-food restaurants and restaurants in the region of Campinas, Brazil, and analyzed using LC-MS/MS	P50 (P97,5)<br>0.04 (0.77)	
**Mixed countries**					
Pelucchi 2017 [101]	International Pancreatic Cancer Case–Control Consortium (PanC4) (*n* = 6214, Cases = 1975, Controls = 4239).	Based on FFQs, each participating study provided information on individual consumption of a number of food items (14-33) together with cooking method whenever relevant.	Data on the average acrylamide content of foods were derived from area-specific resources.	Mean (SD)<br>Site 1, USA, controls: 0.27 (0.17)<br>Site 1, USA, cases: 0.25 (0.16)<br>Site 2, USA, controls: 0.29 (0.20)<br>Site 2, USA, cases : 0.31 (0.18)<br>Site 3, USA, controls: 0.22 (18)<br>Site 3, USA, cases : 0.25 (0.18)<br><br>Australia, controls: 0.26 (0.16)	P50 (P25–75)<br>Site 1, USA, all: 19.0 (12.5–28.0)<br><br>Site 2, USA, all : 17.6 (11.9–26.0)<br><br>Site 3, USA, all: 14.7 (8.3–22.2)<br><br>Site 4, USA, all: 23.1 (16.5–31.9)<br>Australia, all : 17.8 (12.5–25.5)

[56] Same population as [95]

[57] Same population as [95]

	Australia, cases: 0.29 (0.22)	
	Italy, controls: 0.43 (0.24)	Italy, all: 28.2 (19.1–39.6)
	Italy, cases: 0.47 (0.24)	

References

1. Hirvonen, T.P.; Kontto, J.; Jestoi, M.; Valsta, L.M.; Peltonen, K.; Pietinen, P.; Virtanen, S.M.; Sinkko, H.; Kronbergkippila, C.; Albanes, D.; et al. Dietary acrylamide intake and the risk of cancer among Finnish male smokers. *Cancer Causes Control.* **2010**, *21*, 2223–2229, doi:10.1007/s10552-010-9642-2.
2. Hirvonen, T.; Jestoi, M.; Tapanainen, H.; Valsta, L.; Virtanen, S.M.; Sinkko, H.; Kronberg-Kippilä, C.; Kontto, J.; Virtamo, J.; Simell, O.; et al. Dietary acrylamide exposure among Finnish adults and children: The potential effect of reduction measures. *Food Addit. Contam. Part. A* **2011**, *28*, 1483–1491, doi:10.1080/19440049.2011.593559.
3. Dybing, E. Risk Assessment of Acrylamide in Foods. *Toxicol. Sci.* **2003**, *75*, 7–15, doi:10.1093/toxsci/kfg165.
4. Bjellaas, T.; Stølen, L.H.; Haugen, M.; Paulsen, J.E.; Alexander, J.; Lundanes, E.; Becher, G. Urinary acrylamide metabolites as biomarkers for short-term dietary exposure to acrylamide. *Food Chem. Toxicol.* **2007**, *45*, 1020–1026, doi:10.1016/j.fct.2006.12.007.
5. Bjellaas, T.; Olesen, P.T.; Frandsen, H.; Haugen, M.; Stølen, L.H.; Paulsen, J.E.; Alexander, J.; Lundanes, E.; Becher, G. Comparison of Estimated Dietary Intake of Acrylamide with Hemoglobin Adducts of Acrylamide and Glycidamide. *Toxicol. Sci.* **2007**, *98*, 110–117, doi:10.1093/toxsci/kfm091.
6. Brantsæter, A.L.; Haugen, M.; De Mul, A.; Bjellaas, T.; Becher, G.; Van Klaveren, J.; Alexander, J.; Meltzer, H.M. Exploration of different methods to assess dietary acrylamide exposure in pregnant women participating in the Norwegian Mother and Child Cohort Study (MoBa). *Food Chem. Toxicol.* **2008**, *46*, 2808–2814, doi:10.1016/j.fct.2008.05.020.
7. Duarte-Salles, T.; Von Stedingk, H.; Granum, B.; Gützkow, K.B.; Rydberg, P.; Törnqvist, M.; Mendez, M.A.; Brunborg, G.; Brantsæter, A.L.; Meltzer, H.M.; et al. Dietary Acrylamide Intake during Pregnancy and Fetal Growth—Results from the Norwegian Mother and Child Cohort Study (MoBa). *Environ. Health Perspect.* **2013**, *121*, 374–379, doi:10.1289/ehp.1205396.
8. Kadawathagedara, M.; Botton, J.; De Lauzon-Guillain, B.; Meltzer, H.M.; Alexander, J.; Brantsaeter, A.L.; Haugen, M.; Papadopoulou, E. Dietary acrylamide intake during pregnancy and postnatal growth and obesity: Results from the Norwegian Mother and Child Cohort Study (MoBa). *Environ. Int.* **2018**, *113*, 325–334, doi:10.1016/j.envint.2018.01.004.
9. Svensson, K.; Abramsson, L.; Becker, W.; Glynn, A.; Hellenäs, K.-E.; Lind, Y.; Rosén, J. Dietary intake of acrylamide in Sweden. *Food Chem. Toxicol.* **2003**, *41*, 1581–1586, doi:10.1016/s0278-6915(03)00188-1.
10. Mucci, L.A.; Dickman, P.; Steineck, G.; Adami, H.-O.; Augustsson, K. Dietary acrylamide and cancer of the large bowel, kidney, and bladder: Absence of an association in a population-based study in Sweden. *Br. J. Cancer* **2003**, *88*, 84–89, doi:10.1038/sj.bjc.6600726.
11. Mucci, L.A.; Lindblad, P.; Steineck, G.; Adami, H.-O. Dietary acrylamide and risk of renal cell cancer. *Int. J. Cancer* **2004**, *109*, 774–776, doi:10.1002/ijc.20011.
12. Mucci, L.A.; Sandin, S.; Bälter, K.; Adami, H.-O.; Magnusson, C.; Weiderpass, E. Acrylamide Intake and Breast Cancer Risk in Swedish Women. *JAMA* **2005**, *293*, 1322–1327, doi:10.1001/jama.293.11.1326.
13. Mucci, L.A.; Adami, H.-O.; Wolk, A. Prospective study of dietary acrylamide and risk of colorectal cancer among women. *Int. J. Cancer* **2005**, *118*, 169–173, doi:10.1002/ijc.21309.
14. Larsson, S.C.; Åkesson, A.; Wolk, A. Long-term Dietary Acrylamide Intake and Breast Cancer Risk in a Prospective Cohort of Swedish Women. *Am. J. Epidemiol.* **2008**, *169*, 376–381, doi:10.1093/aje/kwn319.
15. Larsson, S.C.; Håkansson, N.; Åkesson, A.; Wolk, A. Long-term dietary acrylamide intake and risk of endometrial cancer in a prospective cohort of Swedish women. *Int. J. Cancer* **2009**, *124*, 1196–1199, doi:10.1002/ijc.24002.
16. Larsson, S.C.; Åkesson, A.; Wolk, A. Long-Term Dietary Acrylamide Intake and Risk of Epithelial Ovarian Cancer in a Prospective Cohort of Swedish Women. *Cancer Epidemiol. Biomark. Prev.* **2009**, *18*, 994–997, doi:10.1158/1055-9965.epi-08-0868.
17. Wirfält, E.; Paulsson, B.; Törnqvist, M.; Axmon, A.; Hagmar, L.; Wirf, E.; Auml], [Associations between estimated acrylamide intakes, and hemoglobin AA adducts in a sample from the Malmö Diet and Cancer cohort. *Eur. J. Clin. Nutr.* **2007**, *62*, 314–323, doi:10.1038/sj.ejcn.1602704.

18. Vikström, A.C.; Warholm, M.; Paulsson, B.; Axmon, A.; Wirfält, E.; Törnqvist, M. Hemoglobin adducts as a measure of variations in exposure to acrylamide in food and comparison to questionnaire data. *Food Chem. Toxicol.* **2012**, *50*, 2531–2539, doi:10.1016/j.fct.2012.04.004.
19. Larsson, S.C.; Åkesson, A.; Bergkvist, L.; Wolk, A. Dietary acrylamide intake and risk of colorectal cancer in a prospective cohort of men. *Eur. J. Cancer* **2009**, *45*, 513–516, doi:10.1016/j.ejca.2008.12.001.
20. Larsson, S.C.; Åkesson, A.; Wolk, A. Dietary Acrylamide Intake and Prostate Cancer Risk in a Prospective Cohort of Swedish Men. *Cancer Epidemiol. Biomark. Prev.* **2009**, *18*, 1939–1941, doi:10.1158/1055-9965.epi-09-0280.
21. Wilson, K.M.; Bälter, K.; Adami, H.-O.; Grönberg, H.; Vikström, A.C.; Paulsson, B.; Törnqvist, M.; Mucci, L.A. Acrylamide exposure measured by food frequency questionnaire and hemoglobin adduct levels and prostate cancer risk in the Cancer of the Prostate in Sweden Study. *Int. J. Cancer* **2009**, *124*, 2384–2390, doi:10.1002/ijc.24175.
22. Lin, Y.; Lagergren, J.; Lu, Y. Dietary acrylamide intake and risk of esophageal cancer in a population-based case-control study in Sweden. *Int. J. Cancer* **2010**, *128*, 676–681, doi:10.1002/ijc.25608.
23. Jakobsen, L.S.; Granby, K.; Knudsen, V.K.; Nauta, M.; Pires, S.M.; Poulsen, M. Burden of disease of dietary exposure to acrylamide in Denmark. *Food Chem. Toxicol.* **2016**, *90*, 151–159, doi:10.1016/j.fct.2016.01.021.
24. Mills, C.; Tlustos, C.; Evans, R.; Matthews, W. Dietary Acrylamide Exposure Estimates for the United Kingdom and Ireland: Comparison between Semiprobabilistic and Probabilistic Exposure Models. *J. Agric. Food Chem.* **2008**, *56*, 6039–6045, doi:10.1021/jf073050x.
25. Burley, V.J.; Greenwood, D.C.; Hepworth, S.J.; Fraser, L.K.; De Kok, T.M.; Van Breda, S.G.; Kyrtopoulos, S.; Botsivali, M.; Kleinjans, J.; McKinney, P.A.; et al. Dietary acrylamide intake and risk of breast cancer in the UK women's cohort. *Br. J. Cancer* **2010**, *103*, 1749–1754, doi:10.1038/sj.bjc.6605956.
26. Konings, E.; Baars, A.; van Klaveren, J.; Spanjer, M.; Rensen, P.; Hiemstra, M.; van Kooij, J.; Peters, P. Acrylamide exposure from foods of the Dutch population and an assessment of the consequent risks. *Food Chem. Toxicol.* **2003**, *41*, 1569–1579, doi:10.1016/s0278-6915(03)00187-x.
27. Boon, P.E.; De Mul, A.; Van Der Voet, H.; Van Donkersgoed, G.; Brette, M.; Van Klaveren, J.D. Calculations of dietary exposure to acrylamide. *Mutat. Res. Toxicol. Environ. Mutagen.* **2005**, *580*, 143–155, doi:10.1016/j.mrgentox.2004.10.014.
28. Hogervorst, J.G.; Schouten, L.; Konings, E.J.; Goldbohm, R.A.; Brandt, P.V.D. Dietary acrylamide intake and the risk of renal cell, bladder, and prostate cancer. *Am. J. Clin. Nutr.* **2008**, *87*, 1428–1438, doi:10.1093/ajcn/87.5.1428.
29. Hogervorst, J.G.F.; Schouten, L.J.; Konings, E.J.M.; Goldbohm, R.A.; Brandt, P.A.V.D. Dietary Acrylamide Intake Is Not Associated with Gastrointestinal Cancer Risk. *J. Nutr.* **2008**, *138*, 2229–2236, doi:10.3945/jn.108.092957.
30. Hogervorst, J.G.; Schouten, L.; Konings, E.J.; Goldbohm, R.A.; Brandt, P.V.D. Dietary Acrylamide Intake and Brain Cancer Risk. *Cancer Epidemiol. Biomark. Prev.* **2009**, *18*, 1663–1666, doi:10.1158/1055-9965.epi-08-1133.
31. Hogervorst, J.G.F.; Schouten, L.; Konings, E.J.M.; Goldbohm, R.A.; Brandt, P.V.D. Lung Cancer Risk in Relation to Dietary Acrylamide Intake. *J. Natl. Cancer Inst.* **2009**, *101*, 651–662, doi:10.1093/jnci/djp077.
32. Hogervorst, J.G.; De Bruijn-Geraets, D.; Schouten, L.J.; Van Engeland, M.; De Kok, T.M.; Goldbohm, R.A.; Brandt, P.A.V.D.; Weijenberg, M.P. Dietary acrylamide intake and the risk of colorectal cancer with specific mutations in KRAS and APC. *Carcinogenesis* **2014**, *35*, 1032–1038, doi:10.1093/carcin/bgu002.
33. Bongers, M.L.; Hogervorst, J.G.F.; Schouten, L.; Goldbohm, R.A.; Schouten, H.C.; Brandt, P.V.D. Dietary Acrylamide Intake and the Risk of Lymphatic Malignancies: The Netherlands Cohort Study on Diet and Cancer. *PLoS ONE* **2012**, *7*, e38016, doi:10.1371/journal.pone.0038016.
34. Lipunova, N.; Schouten, L.; Brandt, P.A.V.D.; Hogervorst, J.G. A prospective cohort study on dietary acrylamide intake and the risk for cutaneous malignant melanoma. *Eur. J. Cancer Prev.* **2017**, *26*, 528–531, doi:10.1097/cej.0000000000000268.
35. Schouten, L.J.; Hogervorst, J.G.F.; Konings, E.J.M.; Goldbohm, R.A.; Brandt, P.V.D. Dietary Acrylamide Intake and the Risk of Head-Neck and Thyroid Cancers: Results from the Netherlands Cohort Study. *Am. J. Epidemiol.* **2009**, *170*, 873–884, doi:10.1093/aje/kwp213.
36. Hogervorst, J.G.; Schouten, L.; Konings, E.J.; Goldbohm, R.A.; Brandt, P.V.D. A Prospective Study of Dietary Acrylamide Intake and the Risk of Endometrial, Ovarian, and Breast Cancer. *Cancer Epidemiol. Biomark. Prev.* **2007**, *16*, 2304–2313, doi:10.1158/1055-9965.epi-07-0581.

37. Pedersen, G.S.; Hogervorst, J.G.F.; Schouten, L.; Konings, E.J.M.; Goldbohm, R.A. (Sandra); Brandt, P.V.D. Dietary acrylamide intake and estrogen and progesterone receptor-defined postmenopausal breast cancer risk. *Breast Cancer Res. Treat.* **2009**, *122*, 199–210, doi:10.1007/s10549-009-0642-4.
38. Hogervorst, J.G.F.; Brandt, P.V.D.; Godschalk, R.W.L.; Van Schooten, F.-J.; Schouten, L. The influence of single nucleotide polymorphisms on the association between dietary acrylamide intake and endometrial cancer risk. *Sci. Rep.* **2016**, *6*, 34902, doi:10.1038/srep34902.
39. Hogervorst, J.G.F.; Brandt, P.V.D.; Godschalk, R.W.L.; Van Schooten, F.-J.; Schouten, L. Interactions between dietary acrylamide intake and genes for ovarian cancer risk. *Eur. J. Epidemiol.* **2017**, *32*, 431–441, doi:10.1007/s10654-017-0244-0.
40. Hogervorst, J.G.F.; Brandt, P.V.D.; Godschalk, R.W.L.; Van Schooten, F.-J.; Schouten, L. Interaction between dietary acrylamide intake and genetic variants for estrogen receptor-positive breast cancer risk. *Eur. J. Nutr.* **2019**, *58*, 1033–1045, doi:10.1007/s00394-018-1619-z.
41. Perloy, A.; Schouten, L.J.; Brandt, P.A.V.D.; Godschalk, R.; Van Schooten, F.-J.; Hogervorst, J.G.F. The Role of Genetic Variants in the Association between Dietary Acrylamide and Advanced Prostate Cancer in the Netherlands Cohort Study on Diet and Cancer. *Nutr. Cancer* **2018**, *70*, 620–631, doi:10.1080/01635581.2018.1460682.
42. Konings, E.J.M.; Hogervorst, J.G.F.; Van Rooij, L.; Schouten, L.; Sizoo, E.A.; Van Egmond, H.P.; Goldbohm, R.A.; Brandt, P.V.D. Validation of a database on acrylamide for use in epidemiological studies. *Eur. J. Clin. Nutr.* **2010**, *64*, 534–540, doi:10.1038/ejcn.2010.17.
43. Matthys, C.; Bilau, M.; Govaert, Y.; Moons, E.; De Henauw, S.; Willems, J. Risk assessment of dietary acrylamide intake in Flemish adolescents. *Food Chem. Toxicol.* **2005**, *43*, 271–278, doi:10.1016/j.fct.2004.10.003.
44. Claeys, W.L.; Baert, K.; Mestdagh, F.; Vercammen, J.; Daenens, P.; De Meulenaer, B.; Maghuin-Rogister, G.; Huyghebaert, A. Assessment of the acrylamide intake of the Belgian population and the effect of mitigation strategies. *Food Addit. Contam. Part. A* **2010**, *27*, 1199–1207, doi:10.1080/19440049.2010.489577.
45. Hilbig, A.; Freidank, N.; Kersting, M.; Wilhelm, M.; Wittsiepe, J. Estimation of the dietary intake of acrylamide by German infants, children and adolescents as calculated from dietary records and available data on acrylamide levels in food groups. *Int. J. Hyg. Environ. Health* **2004**, *207*, 463–471, doi:10.1078/1438-4639-00317.
46. Mojska, H.; Gielecińska, I.; Szponar, L.; Ołtarzewski, M. Estimation of the dietary acrylamide exposure of the Polish population. *Food Chem. Toxicol.* **2010**, *48*, 2090–2096, doi:10.1016/j.fct.2010.05.009.
47. Zając, J.; Bojar, I.; Helbin, J.; Kolarzyk, E.; Potocki, A.; Strzemecka, J.; Owoc, A. Dietary acrylamide exposure in chosen population of South Poland. *Ann. Agric. Environ. Med.* **2013**, *20*, 351–355.
48. Wyka, J.; Tajner-Czopek, A.; Broniecka, A.; Piotrowska, E.; Bronkowska, M.; Biernat, J. Estimation of dietary exposure to acrylamide of Polish teenagers from an urban environment. *Food Chem. Toxicol.* **2015**, *75*, 151–155, doi:10.1016/j.fct.2014.11.003.
49. Mojska, H.; Gielecińska, I.; Zielińska, A.; Winiarek, J.; Sawicki, W. Estimation of exposure to dietary acrylamide based on mercapturic acids level in urine of Polish women post partum and an assessment of health risk. *J. Expo. Sci. Environ. Epidemiol.* **2015**, *26*, 288–295, doi:10.1038/jes.2015.12.
50. Sirot, V.; Hommet, F.; Tard, A.; Leblanc, J.-C. Dietary acrylamide exposure of the French population: Results of the second French Total Diet Study. *Food Chem. Toxicol.* **2012**, *50*, 889–894, doi:10.1016/j.fct.2011.12.033.
51. Mancini, F.R.; Sirot, V.; Busani, L.; Volatier, J.-L.; Hulin, M. Use and impact of usual intake models on dietary exposure estimate and risk assessment of chemical substances: A practical example for cadmium, acrylamide and sulphites. *Food Addit. Contam. Part. A* **2015**, *32*, 1065–1074, doi:10.1080/19440049.2015.1041428.
52. Chan-Hon-Tong, A.; Charles, M.A.; Forhan, A.; Heude, B.; Sirot, V. Exposure to food contaminants during pregnancy. *Sci. Total. Environ.* **2013**, *458-460*, 27–35, doi:10.1016/j.scitotenv.2013.03.100.
53. Kadawathagedara, M.; Tong, A.C.H.; Heude, B.; Forhan, A.; Charles, M.A.; Sirot, V.; Botton, J.; The EDEN mother-child cohort study group Dietary acrylamide intake during pregnancy and anthropometry at birth in the French EDEN mother-child cohort study. *Environ. Res.* **2016**, *149*, 189–196, doi:10.1016/j.envres.2016.05.019.
54. Pelucchi, C.; Galeone, C.; Levi, F.; Negri, E.; Franceschi, S.; Talamini, R.; Bosetti, C.; Giacosa, A.; La Vecchia, C. Dietary acrylamide and human cancer. *Int. J. Cancer* **2006**, *118*, 467–471, doi:10.1002/ijc.21336.
55. Pelucchi, C.; Galeone, C.; Maso, L.D.; Talamini, R.; Montella, M.; Ramazzotti, V.; Negri, E.; Franceschi, S.; La Vecchia, C. Dietary acrylamide and renal cell cancer. *Int. J. Cancer* **2006**, *120*, 1376–1377, doi:10.1002/ijc.22457.

56. Pelucchi, C.; Galeone, C.; Talamini, R.; Negri, E.; Polesel, J.; Serraino, D.; La Vecchia, C. Dietary acrylamide and pancreatic cancer risk in an Italian case–control study. *Ann. Oncol.* **2011**, *22*, 1910–1915, doi:10.1093/annonc/mdq672.
57. Pelucchi, C.; Galeone, C.; Negri, E.; Bosetti, C.; Serraino, D.; Montella, M.; Talamini, R.; La Vecchia, C. Dietary acrylamide and the risk of endometrial cancer: An Italian case-control study. *Nutr. Cancer* **2016**, *68*, 1–6, doi:10.1080/01635581.2016.1142585.
58. Kafouris, D.; Stavroulakis, G.; Christofidou, M.; Iakovou, X.; Christou, E.; Paikousis, L.; Christodoulidou, M.; Ioannou-Kakouri, E.; Yiannopoulos, S. Determination of acrylamide in food using a UPLC–MS/MS method: Results of the official control and dietary exposure assessment in Cyprus. *Food Addit. Contam. Part. A* **2018**, *35*, 1928–1939, doi:10.1080/19440049.2018.1508893.
59. Obón-Santacana, M.; Slimani, N.; Lujan-Barroso, L.; Travier, N.; Hallmans, G.; Freisling, H.; Ferrari, P.; Boutron-Ruault, M.C.; Racine, A.; Clavel, F.; et al. Dietary intake of acrylamide and pancreatic cancer risk in the European Prospective Investigation into Cancer and Nutrition (EPIC) cohort. *Ann. Oncol.* **2013**, *24*, 2645–2651, doi:10.1093/annonc/mdt255.
60. Freisling, H.; Moskal, A.; Ferrari, P.; Nicolas, G.; Knaze, V.; Clavel-Chapelon, F.; Boutron-Ruault, M.-C.; Nailler, L.; Teucher, B.; Grote, V.A.; et al. Dietary acrylamide intake of adults in the European Prospective Investigation into Cancer and Nutrition differs greatly according to geographical region. *Eur. J. Nutr.* **2012**, *52*, 1369–1380, doi:10.1007/s00394-012-0446-x.
61. Ferrari, P.; Freisling, H.; Duell, E.J.; Kaaks, R.; Lujan-Barroso, L.; Clavel-Chapelon, F.; Boutron-Ruault, M.-C.; Nailler, L.; Polidoro, S.; Mattiello, A.; et al. Challenges in estimating the validity of dietary acrylamide measurements. *Eur. J. Nutr.* **2012**, *52*, 1503–1512, doi:10.1007/s00394-012-0457-7.
62. Obón-Santacana, M.; Kaaks, R.; Slimani, N.; Lujan-Barroso, L.; Freisling, H.; Ferrari, P.; Dossus, L.; Chabbert-Buffet, N.; Baglietto, L.; Fortner, R.; et al. Dietary intake of acrylamide and endometrial cancer risk in the European Prospective Investigation into Cancer and Nutrition cohort. *Br. J. Cancer* **2014**, *111*, 987–997, doi:10.1038/bjc.2014.328.
63. Lujan-Barroso, L.; González, C.A.; Slimani, N.; Obón-Santacana, M.; Ferrari, P.; Freisling, H.; Overvad, K.; Clavel-Chapelon, F.; Boutron-Ruault, M.-C.; Racine, A.; et al. Dietary intake of acrylamide and esophageal cancer risk in the European Prospective Investigation into Cancer and Nutrition cohort. *Cancer Causes Control.* **2014**, *25*, 639–646, doi:10.1007/s10552-014-0359-5.
64. Obón-Santacana, M.; Peeters, P.H.; Freisling, H.; Dossus, L.; Clavel-Chapelon, F.; Baglietto, L.; Schock, H.; Fortner, R.; Boeing, H.; Tjonneland, A.; et al. Dietary Intake of Acrylamide and Epithelial Ovarian Cancer Risk in the European Prospective Investigation into Cancer and Nutrition (EPIC) Cohort. *Cancer Epidemiol. Biomark. Prev.* **2015**, *24*, 291–297, doi:10.1158/1055-9965.epi-14-0636.
65. Obón-Santacana, M.; Lujan-Barroso, L.; Freisling, H.; Cadeau, C.; Fagherazzi, G.; Boutron-Ruault, M.-C.; Kaaks, R.; Fortner, R.; Boeing, H.; Quirós, J.R.; et al. Dietary and lifestyle determinants of acrylamide and glycidamide hemoglobin adducts in non-smoking postmenopausal women from the EPIC cohort. *Eur. J. Nutr.* **2016**, *56*, 1157–1168, doi:10.1007/s00394-016-1165-5.
66. Cengiz, M.F.; Gündüz, C.P.B. Acrylamide exposure among Turkish toddlers from selected cereal-based baby food samples. *Food Chem. Toxicol.* **2013**, *60*, 514–519, doi:10.1016/j.fct.2013.08.018.
67. Nematollahi, A.; Kamankesh, M.; Hosseini, H.; Ghasemi, J.; Hosseini-Esfahani, F.; Mohammadi, A.; Khaneghah, A.M. Acrylamide content of collected food products from Tehran's market: A risk assessment study. *Environ. Sci. Pollut. Res.* **2020**, *27*, 30558–30570, doi:10.1007/s11356-020-09323-w.
68. Zhou, P.P.; Zhao, Y.F.; Liu, H.L.; Ma, Y.J.; Li, X.W.; Yang, X.; Wu, Y. Dietary exposure of the Chinese population to acrylamide. *Biomed. Environ. Sci.* **2013**, *26*, doi:10.3967/0895-3988.2013.06.002.
69. Gao, J.; Zhao, Y.; Zhu, F.; Ma, Y.; Li, X.; Miao, H.; Wu, Y. Dietary exposure of acrylamide from the fifth Chinese Total Diet Study. *Food Chem. Toxicol.* **2016**, *87*, 97–102, doi:10.1016/j.fct.2015.11.013.
70. Chen, F.; Yuan, Y.; Liu, J.; Zhao, G.; Hu, X. Survey of acrylamide levels in Chinese foods. *Food Addit. Contam. Part. B* **2008**, *1*, 85–92, doi:10.1080/02652030802512461.
71. Wong, W.W.; Chung, S.W.; Lam, C.-H.; Ho, Y.; Xiao, Y. Dietary exposure of Hong Kong adults to acrylamide: Results of the first Hong Kong Total Diet Study. *Food Addit. Contam. Part. A* **2014**, *31*, 799–805, doi:10.1080/19440049.2014.898189.
72. Liu, Z.-M.; Tse, L.A.; Ho, S.C.; Wu, S.; Chen, B.; Chan, D.; Wong, S.Y.-S. Dietary acrylamide exposure was associated with increased cancer mortality in Chinese elderly men and women: A 11-year prospective study of Mr. and Ms. OS Hong Kong. *J. Cancer Res. Clin. Oncol.* **2017**, *143*, 2317–2326, doi:10.1007/s00432-017-2477-4.

73. Liu, Z.-M.; Tse, L.A.; Chen, B.; Wu, S.; Chan, D.; Kowk, T.; Woo, J.; Xiang, Y.-T.; Wong, S.Y.-S. Dietary acrylamide exposure was associated with mild cognition decline among non-smoking Chinese elderly men. *Sci. Rep.* **2017**, *7*, 1–7, doi:10.1038/s41598-017-06813-9.
74. Lee, S.; Kim, H.J. Dietary Exposure to Acrylamide and Associated Health Risks for the Korean Population. *Int. J. Environ. Res. Public Health* **2020**, *17*, 7619, doi:10.3390/ijerph17207619.
75. Jeong, H.; Hwang, S.; Kwon, H. Survey for acrylamide in processed foods from Korean market and individual exposure estimation using a non-parametric probabilistic model. *Food Addit. Contam. Part. A* **2020**, *37*, 916–930, doi:10.1080/19440049.2020.1746410.
76. Nagata, C.; Konishi, K.; Tamura, T.; Wada, K.; Tsuji, M.; Hayashi, M.; Takeda, N.; Yasuda, K. Associations of Acrylamide Intake with Circulating Levels of Sex Hormones and Prolactin in Premenopausal Japanese Women. *Cancer Epidemiol. Biomark. Prev.* **2015**, *24*, 249–254, doi:10.1158/1055-9965.epi-14-0935.
77. Acrylamide Intake with Urinary Sex Hormone Levels among Preschool Japanese Children. *Am. J. Epidemiol.* **2017**, *187*, 75–81, doi:10.1093/aje/kwx197.
78. Kawahara, J.; Imaizumi, Y.; Kuroda, K.; Aoki, Y.; Suzuki, N. Estimation of long-term dietary exposure to acrylamide of the Japanese people. *Food Addit. Contam. Part. A* **2018**, *35*, 1689–1702, doi:10.1080/19440049.2018.1484179.
79. Yamamoto, J.; Ishihara, J.; Kotemori, A.; Nakadate, M.; Sobue, T. Validity of Estimated Acrylamide Intake by the Dietary Record Method and Food Frequency Questionnaire in Comparison with a Duplicate Method: A Pilot Study. *J. Nutr. Sci. Vitaminol.* **2018**, *64*, 340–346, doi:10.3177/jnsv.64.340.
80. Kotemori, A.; Ishihara, J.; Nakadate, M.; Sawada, N.; Iwasaki, M.; Sobue, T.; Tsugane, S. Validity of a Self-administered Food Frequency Questionnaire for the Estimation of Acrylamide Intake in the Japanese Population: The JPHC FFQ Validation Study. *J. Epidemiol.* **2018**, *28*, 482–487, doi:10.2188/jea.je20170186.
81. Kotemori, A.; Ishihara, J.; Zha, L.; Liu, R.; Sawada, N.; Iwasaki, M.; Sobue, T.; Tsugane, S.; The JPHC Study Group Dietary acrylamide intake and the risk of endometrial or ovarian cancers in Japanese women. *Cancer Sci.* **2018**, *109*, 3316–3325, doi:10.1111/cas.13757.
82. Kotemori, A.; Ishihara, J.; Zha, L.; Liu, R.; Sawada, N.; Iwasaki, M.; Sobue, T.; Tsugane, S.; The JPHC Study Group Dietary acrylamide intake and risk of breast cancer: The Japan Public Health Center-based Prospective Study. *Cancer Sci.* **2017**, *109*, 843–853, doi:10.1111/cas.13496.
83. Liu, R.; Sobue, T.; Kitamura, T.; Kitamura, Y.; Ishihara, J.; Kotemori, A.; Zha, L.; Ikeda, S.; Sawada, N.; Iwasaki, M.; et al. Dietary Acrylamide Intake and Risk of Esophageal, Gastric, and Colorectal Cancer: The Japan Public Health Center-based Prospective Study. *Cancer Epidemiol. Biomark. Prev.* **2019**, *28*, 1461–1468, doi:10.1158/1055-9965.epi-18-1259.
84. Nagata, C.; Konishi, K.; Wada, K.; Tamura, T.; Goto, Y.; Koda, S.; Mizuta, F.; Iwasa, S. Maternal Acrylamide Intake during Pregnancy and Sex Hormone Levels in Maternal and Umbilical Cord Blood and Birth Size of Offspring. *Nutr. Cancer* **2018**, *71*, 77–82, doi:10.1080/01635581.2018.1524018.
85. Tong, P.; Fuentes, D.; Hambridge, T. Australian survey of acrylamide in carbohydrate-based foods. *Food Addit. Contam.* **2004**, *21*, 721–736, doi:10.1080/02652030412331272458.
86. Normandin, L.; Bouchard, M.; Ayotte, P.; Blanchet, C.; Becalski, A.; Bonvalot, Y.; Phaneuf, D.; Lapointe, C.; Gagné, M.; Courteau, M. Dietary exposure to acrylamide in adolescents from a Canadian urban center. *Food Chem. Toxicol.* **2013**, *57*, 75–83, doi:10.1016/j.fct.2013.03.005.
87. Brisson, B.; Ayotte, P.; Normandin, L.; Gaudreau, E.; Bienvenu, J.-F.; Fennell, T.R.; Blanchet, C.; Phaneuf, D.; Lapointe, C.; Bonvalot, Y.; et al. Relation between dietary acrylamide exposure and biomarkers of internal dose in Canadian teenagers. *J. Expo. Sci. Environ. Epidemiol.* **2013**, *24*, 215–221, doi:10.1038/jes.2013.34.
88. Wilson, K.M.; Vesper, H.W.; Tocco, P.; Sampson, L.; Rosén, J.; Hellenäs, K.-E.; Törnqvist, M.; Willett, W.C. Validation of a food frequency questionnaire measurement of dietary acrylamide intake using hemoglobin adducts of acrylamide and glycidamide. *Cancer Causes Control.* **2009**, *20*, 269–278, doi:10.1007/s10552-008-9241-7.
89. Wilson, K.M.; Mucci, L.A.; Cho, E.; Hunter, D.J.; Chen, W.Y.; Willett, W.C. Dietary Acrylamide Intake and Risk of Premenopausal Breast Cancer. *Am. J. Epidemiol.* **2009**, *169*, 954–961, doi:10.1093/aje/kwn421.
90. Wilson, K.M.; Mucci, L.A.; Rosner, B.A.; Willett, W.C. A Prospective Study on Dietary Acrylamide Intake and the Risk for Breast, Endometrial, and Ovarian Cancers. *Cancer Epidemiol. Biomark. Prev.* **2010**, *19*, 2503–2515, doi:10.1158/1055-9965.epi-10-0391.
91. Hogervorst, J.G.; Fortner, R.; Mucci, L.A.; Tworoger, S.; Eliassen, A.H.; Hankinson, S.E.; Wilson, K.M. Associations between Dietary Acrylamide Intake and Plasma Sex Hormone Levels. *Cancer Epidemiol. Biomark. Prev.* **2013**, *22*, 2024–2036, doi:10.1158/1055-9965.epi-13-0509.
92. Graff, R.E.; Cho, E.; Preston, M.A.; Sanchez, A.; Mucci, L.A.; Wilson, K.M. Dietary Acrylamide Intake and Risk of Renal Cell Carcinoma in Two Large Prospective Cohorts. *Cancer Epidemiol. Biomark. Prev.* **2018**, *27*, 979–982, doi:10.1158/1055-9965.epi-18-0320.
93. Wilson, K.M.; Giovannucci, E.; Stampfer, M.J.; Mucci, L.A. Dietary acrylamide and risk of prostate cancer. *Int. J. Cancer* **2011**, *131*, 479–487, doi:10.1002/ijc.26383.

94. Doerge, D.R.; Young, J.F.; Chen, J.J.; DiNovi, M.J.; Henry, S.H. Using Dietary Exposure and Physiologically Based Pharmacokinetic/Pharmacodynamic Modeling in Human Risk Extrapolations for Acrylamide Toxicity. *J. Agric. Food Chem.* **2008**, *56*, 6031–6038, doi:10.1021/jf073042g.
95. Tran, N.; Barraj, L.; Murphy, M.; Bi, X. Dietary Acrylamide Exposure and Hemoglobin Adducts—National Health and Nutrition Examination Survey (2003–04). *Food Chem. Toxicol.* **2010**, *48*, 3098–3108, doi:10.1016/j.fct.2010.08.003.
96. Katz, J.M.; Winter, C.K.; Buttrey, S.E.; Fadel, J.G. Comparison of acrylamide intake from Western and guideline based diets using probabilistic techniques and linear programming. *Food Chem. Toxicol.* **2012**, *50*, 877–883, doi:10.1016/j.fct.2011.11.006.
97. Duke, T.J.; Ruestow, P.S.; Marsh, G.M. The influence of demographic, physical, behavioral, and dietary factors on hemoglobin adduct levels of acrylamide and glycidamide in the general U.S. population. *Crit. Rev. Food Sci. Nutr.* **2017**, *58*, 700–710, doi:10.1080/10408398.2016.1215289.
98. Abt, E.; Robin, L.P.; McGrath, S.; Srinivasan, J.; DiNovi, M.; Adachi, Y.; Chirtel, S. Acrylamide levels and dietary exposure from foods in the United States, an update based on 2011-2015 data. *Food Addit. Contam. Part. A* **2019**, *36*, 1475–1490, doi:10.1080/19440049.2019.1637548.
99. McCullough, M.L.; Hodge, R.A.; Um, C.Y.; Gapstur, S.M. Dietary Acrylamide Is Not Associated with Renal Cell Cancer Risk in the CPS-II Nutrition Cohort. *Cancer Epidemiol. Biomark. Prev.* **2019**, *28*, 616–619, doi:10.1158/1055-9965.epi-18-0909.
100. Arisseto, A.P.T.; de Figueiredo, M.C.; Govaert, Y.; van Loco, J.; Fraselle, S.; Degroodt, J.-M.; Rosseto Caroba, D.C. Contribution of selected foods to acrylamide intake by a population of Brazilian adolescents. *Food Sci. Technol.* **2009**, *42*, 207–211.
101. Pelucchi, C.; Rosato, V.; Bracci, P.M.; Li, D.; Neale, R.E.; Lucenteforte, E.; Serraino, D.; Anderson, K.E.; Fontham, E.; Holly, E.A.; et al. Dietary acrylamide and the risk of pancreatic cancer in the International Pancreatic Cancer Case–Control Consortium (PanC4). *Ann. Oncol.* **2017**, *28*, 408–414, doi:10.1093/annonc/mdw618.
